# Cardamonin attenuates SH-SY5Y neuronal apoptosis induced by LPS-activated microglia via modulation of the EGFR/PI3K/AKT1/ERK/NF-κB pathway

**DOI:** 10.3389/fphar.2026.1832453

**Published:** 2026-10-19

**Authors:** Jingjing Lu, Rong Zhou, Kechao Tang, Hangwei Wang, Zhongxia Shen, Chao Huang, Lijun Cui

**Affiliations:** 1 Key Laboratory of Psychiatry, Huzhou Third Municipal Hospital, The Affiliated Hospital of Huzhou Normal University, Huzhou, China; 2 Sleep Medical Center, Huzhou Third Municipal Hospital, The Affiliated Hospital of Huzhou University, Huzhou, China; 3 Department of Pharmacology, School of Pharmacy, Nantong University, Nantong, China

**Keywords:** BV2 cells, cardamonin, EGFR/PI3K/AKT1 signaling, network pharmacology, neurodegenerative diseases, neuroinflammation, NF-κB signaling, SH-SY5Y cells

## Abstract

**Introduction:**

Microglial activation-induced neuroinflammation plays a critical role in the pathogenesis of neurodegenerative diseases. Cardamonin (CD, 2′,4′-dihydroxy-6′-methoxychalcone), a natural chalcone flavonoid present in several medicinal plants, demonstrates significant anti-inflammatory activity. Nevertheless, the precise mechanisms by which it mitigates neuroinflammation remain incompletely understood.

**Methods:**

In this study, the potential targets and pathways of CD were investigated using network pharmacology and molecular docking. The neuroinflammatory protective mechanisms were examined *in vitro* using LPS-stimulated BV2 cells and a BV2/SH-SY5Y co-culture system, employing ELISA, RT-qPCR, Western blot, immunofluorescence, and other assays.

**Results and Discussion:**

Network pharmacology identified epidermal growth factor receptor (EGFR), rac-alpha serine/threonine-protein kinase (AKT1), heat shock protein 90 alpha family class B member 1 (HSP90AB1), prostaglandin-endoperoxide synthase 2 (PTGS2/COX-2) and transcription factor p65 (RELA) as key potential targets, with the EGFR/PI3K/AKT1, MAPK/ERK, and NF-κB signaling pathways being the main enriched pathways. In BV2 cells, CD suppressed LPS-induced production of IL-6, IL-1β, TNF-α, and nitric oxide (NO), and modulated the EGFR/PI3K/AKT1/ERK/NF-κB signaling axis. Pharmacological inhibition of EGFR or PI3K phenocopied the effects of CD, supporting the functional involvement of this pathway. Additionally, CD attenuated reactive oxygen species (ROS) accumulation and prevented the loss of mitochondrial membrane potential. In a co-culture model, CD pretreatment reduced BV2-conditioned medium neurotoxicity toward SH-SY5Y neurons. Collectively, these findings suggest that CD alleviates neuroinflammation through modulation of the EGFR/PI3K/AKT1/ERK/NF-κB pathway, mitigating inflammatory and oxidative stress responses and providing indirect neuroprotection, supporting its potential application in neuroinflammation-associated neurodegenerative diseases.

## 1 Introduction

Neuroinflammation is a significant factor in the pathological mechanisms of various neurodegenerative diseases (Zhang et al., 2023). As the primary resident immune cells of the central nervous system, microglia serve as the first line of defense against pathological insults (Streit et al., 2005; Nakajima and Kohsaka, 2004). Upon exposure to alterations in the microenvironment or pathological stimuli, microglia become activated and undergo morphological and phenotypic changes, establishing their essential function in neuroimmune regulation (Liu and Hong, 2003). Microglial activation, induced by various pathological stimuli such as lipopolysaccharide (LPS), interferon-γ (IFN-γ), and β-amyloid (Aβ) (Subhramanyam et al., 2019), leads to increased production of interleukin-6 (IL-6), tumor necrosis factor-α (TNF-α), interleukin-1β (IL-1β), nitric oxide (NO), and multiple proteolytic enzymes (Block and Hong, 2005). The overproduction of these inflammatory molecules is strongly associated with neuronal injury and synaptic dysfunction, playing a critical role in the progression of neurodegenerative diseases (Stephenson et al., 2018). Therefore, targeting microglia- mediated neuroinflammatory processes represents a promising therapeutic approach for neuroprotection and holds considerable potential in the development of treatments for neurological disorders.

Over recent years, there has been growing interest in plant-derived natural substances with neuroprotective and anti-inflammatory potential (Fakhri et al., 2021; Moise et al., 2024; Nishal et al., 2023). These naturally occurring compounds are generally abundant, inexpensive, and possess a well-established record of safe application across traditional medicinal systems. Cardamonin (CD, 2′,4′-dihydroxy-6′-methoxychalcone), a naturally occurring chalcone flavonoid, is abundant in several traditional medicinal plants. Recent studies have revealed that CD possesses a variety of pharmacological properties, such as anti-inflammatory, antioxidant, antitumor, and vasodilatory activities (Nawaz et al., 2020; Gonçalves et al., 2014; Peng et al.; Chow et al., 2012). Earlier studies have established the neuroprotective potential of CD in animal models. In 5XFAD transgenic mice, CD effectively alleviated cognitive impairment through dual suppression of neuroinflammatory responses and apoptotic processes (Smail, 2024). In a mouse model of cerebral ischemia/reperfusion injury, CD activated the HIF-1α/VEGFA pathway to protect against brain injury (Ni et al., 2022). In a rat model of sciatic nerve crush injury, CD promoted motor and sensory recovery through anti-inflammatory, neurotrophic, and anti-apoptotic effects (Hasan et al., 2025). *In vitro* studies indicated that the neuroprotective mechanism of CD primarily involves inhibition of NF-κB signaling pathway activation in microglial cells, leading to reduced production of pro-inflammatory mediators (Chow et al., 2012; Barber et al., 2023). Additionally, other research has shown that CD could activate the Nrf2-dependent antioxidant defense system, enhance the expression of key antioxidant enzymes, and thereby alleviate neuronal damage caused by oxidative stress (Peng et al., 2017). The neuroinflammatory protective effect of CD has been reported as a key factor in the potential mechanism of its pharmacological functions. However, limited studies regarding this have been conducted and the molecular mechanism remains unclear.

The present work initially performed network pharmacology for exploring the potential mechanisms of the neuroprotective effect of CD. Analysis of protein-protein interaction (PPI) network identified 11 main targets of CD on neurodegenerative disease, including epidermal growth factor receptor (EGFR), rac-alpha serine/threonine-protein kinase (AKT1), heat shock protein 90 alpha family class B member 1 (HSP90AB1), estrogen receptor 1 (ESR1), protein kinase A catalytic subunit alpha (PRKACA), prostaglandin-endoperoxide synthase 2 (PTGS2/COX-2), microtubule-associated protein tau (MAPT), cyclin-dependent kinase 1 (CDK1), peroxisome proliferator-activated receptor gamma (PPARG), transcription factor p65 (RELA), and histone deacetylase 1 (HDAC1). Additionally, analyses using Gene Ontology (GO) and Kyoto Encyclopedia of Genes and Genomes (KEGG) revealed significant enrichment of inflammation-related pathways, including EGFR, PI3K/AKT1and MAPK. These findings imply that CD may influence the regulation of inflammatory responses in the treatment of neurodegenerative disorders. Molecular docking studies further suggested potential interactions between CD and key proteins within the EGFR/PI3K/AKT1, MAPK and NF-κB signaling pathways, such as EGFR, AKT1, HSP90AB1, IKKB, RELA (NF-κB p65), PTGS2, and MAPK1. EGFR, a receptor tyrosine kinase, plays a critical role in regulating cell proliferation, survival, migration and differentiation (Oda et al., 2005). Previously, it has been found that neuroinflammation could activate EGFR in human or mouse microglia cells, thereby activating the downstream PI3K/AKT and ERK pathways to induce inflammatory responses (Lv et al., 2021; Dai et al., 2025). Additionally, the activation of ERK and PI3K/AKT signaling could result in the stimulation of the transcription factor NF-κB (Zheng et al., 2023). Based on these observations, we hypothesized that CD could ameliorate neuroinflammation, and the effect may involve inhibition of the EGFR/PI3K/AKT1/ERK/NF-κB signaling pathways.

Based on network pharmacology predictions, this study investigated the effect of CD on neuroinflammation using an LPS-induced BV2 cell model. Furthermore, the protective role of CD in neurons was explored through a BV2 and SH-SY5Y co-culture system by mitigating neuroinflammation. These findings may contribute to a better understanding of the mechanisms underlying the neuroprotective effects of CD.

## 2 Materials and methods

### 2.1 Potential targets prediction

To identify the potential targets of CD, several databases were utilized, including the Traditional Chinese Medicine Systems Pharmacology Database and Analysis Platform (TCMSP), (http://tcmspw.com/tcmsp.php), the Swiss Target Prediction platform (http://www.swisstargetprediction.ch/), and the Bioinformatics Analysis Tool for Molecular Mechanism of Traditional Chinese Medicine (BATMAN-TCM) (http://bionet.ncpsb.org/batman-tcm/). Targets associated with neurodegenerative diseases were then retrieved from the GeneCards (https://www.genecards.org/) and OMIM (https://www.omim.org/) databases. For GeneCards, all entries with a relevance score >1 were retrieved according to the database’s standard usage paradigm, which prioritizes broad recall followed by subsequent intersection convergence (Stelzer et al., 2016). The specificity of the final target set was achieved through intersection with compound-derived targets rather than through a stringent cutoff at the GeneCards stage. All identified targets were subsequently standardized and validated using the UniProt database (https://www.uniprot.org/), with *Homo sapiens* selected as the target species. Specifically, for SwissTargetPrediction, CD (PubChem CID 641785) was queried against the *Homo sapiens* model and all 100 returned predicted targets were retained, in accordance with the database’s standard usage in network-pharmacology applications, where the probability score reflects relative ranking rather than a hard binary cutoff (Daina et al., 2019). For TCMSP, the CD entry was queried and only Drug Bank-curated experimentally validated targets were retained (Ru et al., 2014); entries with missing HGNC gene symbols were discarded, yielding 28 targets. For BATMAN-TCM, ingredient 67293 was queried and the cluster-1 high-confidence cutoff Score ≥2 (the database’s recommended threshold) was applied (Liu et al., 2016), yielding 44 targets. Targets from the three databases were then mapped to official HGNC symbols and de-duplicated by gene symbol, with the source field concatenated to retain provenance, producing the non-redundant set of 158 CD targets described in Section 3.1.

### 2.2 PPI network establishment

A PPI network was constructed using the STRING database (https://cn.string-db.org/) for *Homo sapiens*, with an interaction score threshold set at high confidence (>0.700). The network was subsequently visualized and analyzed using Cytoscape 3.7.2.

### 2.3 GO and KEGG pathway enrichment analysis

GO and KEGG enrichment analyses were conducted using the Database for Annotation, Visualization, and Integrated Discovery (DAVID) Knowledgebase, version 2021q4 (accessible at https://david-d.ncifcrf.gov/) with *Homo sapiens* as the background species, and results with a false discovery rate (FDR)-adjusted *P* < 0.05 were considered statistically significant.

### 2.4 Molecular docking

To validate the predictions from network pharmacology, molecular docking was conducted. The protein structures were obtained from RCSB Protein Data Bank (https://www.rcsb.org/), and the corresponding ligand-binding sites were identified using DoGSiteScorer of Proteins Plus (https://proteins.plus/). Docking simulations were conducted using LeDock software. A binding energy cutoff of −5.0 kcal/mol was adopted as a criterion for evaluating the reliability of predicted interactions, with lower values indicating stronger binding affinity and greater thermodynamic stability of the resulting ligand-protein complexes. Visualization and analysis of molecular interactions were carried out using Discovery Studio Visualizer 2021.

### 2.5 Cell culture and conditioned medium (CM) preparation

The BV2 mouse microglial cell line (Cat: SCSP-5208) and the SH-SY5Y human neuroblastoma cell line (Cat: SCSP-5014) were sourced from the Shanghai Institute of Cell Biology, Chinese Academy of Sciences (Shanghai, China). Cell cultivation was conducted using Dulbecco’s Modified Eagle Medium (DMEM, Cat: PM150210, Pricella, Wuhan, China), which was enriched with 10% fetal bovine serum (FBS, Cat: C0235, Gibco, Waltham, MA, USA) and 1% penicillin-streptomycin solution (Cat: PB180120, Pricella, Wuhan, China). All cells were cultured in a humidified 5% CO_2_ at a constant temperature of 37 °C.

CD was dissolved in DMSO as a 10 mM stock solution. The control had a concentration of DMSO of no more than 0.16%. During the preparation of CM, BV2 cells were seeded into 6-well plates at a density of 1 × 10^5^ cells per well and serum-starved before treatment. The cells were pretreated with CD at final concentrations of 4, 8, and 16 μM for 2 h, followed by stimulation with 1 μg/mL LPS (Cat: L2880-100 MG, Sigma-Aldrich, USA) for 24 h, A separate group was treated with CD alone at 16 μM for a corresponding duration without LPS stimulation. After treatment, the medium was collected and centrifuged (150 × g, 5 min) to remove cellular debris. The resulting supernatant, referred to as CM from BV2 cells, was stored at −20 °C until further use. Prior to transfer to SH-SY5Y cell cultures, the levels of pro-inflammatory mediators in the CM were evaluated.

### 2.6 Cell viability assay

BV2 cells were plated in 96-well plates at a density of 5 × 10^4^ cells per well and cultured for 24 h. Subsequently, they were treated with gradient concentrations of CD (2, 4, 8, 10, 16, 20, 30 and 40 μM) or LPS (0.25, 0.5, 1, 2, and 4 μg/mL) for further 24 h. Control cells were treated with DMSO. Following the treatment, cell viability was evaluated using a cell count kit-8 (CCK-8, Cat: P-CA-001, Pricella, Wuhan, China). The absorbance at 450 nm was measured using a spectrophotometer (Thermo Scientific Multiskan Skylitch, Type 1550, USA). Separately, SH-SY5Y cells (5 × 10^3^ cells/well) were seeded in 96-well plates and treated with CM for 24 h, and cell viability was determined employing the identical CCK-8 protocol described above.

### 2.7 Assessment of morphological changes

BV2 microglial cells (1 × 10^5^ cells/well) were incubated overnight in 6-well plates at 37 °C.The cells were then pretreated with CD at final concentrations of 4, 8, and 16 μM for 2 h, and then stimulation with LPS (1 μg/mL) for 24 h. Control cells were treated with DMSO. The morphological changes were observed and photographed using EOS Utility Software (Canon USA, Inc). SH-SY5Y cells, seed at a density of 3.5 × 10^5^ cells per well, were exposed to CM from each BV2 cell group. The morphological changes of SH-SY5Y cells were subsequently photographed using the same software.

### 2.8 RNA extraction and real-time quantitative PCR (RT-qPCR)

Total RNA was isolated from pretreated BV2 cells with an RNA-Quick Purification Kit (Cat: RN001, ESscience Biotech, Shanghai, China). First-strand cDNA was synthesized using a First Strand cDNA Synthesis Kit (Cat: D7168S, Beyotime, Shanghai, China). RT-qPCR was conducted on an Applied Biosystems 7500 system (Thermo Fisher Scientific, Waltham, MA, USA) with SYBR Green Master Mix (Cat: A25742, Thermo Fisher, MA, USA) to quantify target gene expression, normalized to glyceraldehyde 3-phosphate de hydrogenase (GAPDH) and analyzed via the ^ΔΔ^CT method. Primer sequences are listed in Table 1.

**TABLE 1:** Primers for RT-qPCR.

Gene	​	Sequences
EGFR	Forward primer	GCC​ATC​TGG​GCC​AAA​GAT​ACC
	Reverse primer	GTC​TTC​GCA​TGA​ATA​GGC​CAA​T
PIK3CA	Forward primer	TAT​GTC​TAC​CCT​CCA​AAT​GTC​G
	Reverse primer	TAC​TTC​TGC​TTG​TCG​TTG​TTT​G
AKT1	Forward primer	GAG​GCA​GGA​AGA​AGA​GAC​GAT​GG
	Reverse primer	ACA​CGG​TGC​TTG​GGC​TTG​G
NF-κB	Forward primer	ATG​GCA​GAC​GAT​GAT​CCC​TAC
	Reverse primer	CGG​AAT​CGA​AAT​CCC​CTC​TGT​T
Iκ-Bα	Forward primer	TGA​AGG​ACG​AGG​AGT​ACG​AGC
	Reverse primer	TGC​AGG​AAC​GAG​TCT​CCG​T
IL-6	Forward primer	AGT​TGC​CTT​CTT​GGG​ACT​GA
	Reverse primer	TTC​TGC​AAG​TGC​ATC​ATC​GT
IL-1β	Forward primer	CAA ATC TCG CAG CAG CAC ATC
	Reverse primer	TCA TCT CGG AGC CTG TAG TGC
TNF-α	Forward primer	CGA​GTG​ACA​AGC​CTG​TAG​CCC
	Reverse primer	GGG​CAG​CCT​TGT​CCC​TTG​A
iNOS	Forward primer	CAT​GCT​ACT​GGA​GGT​GGG​TG
	Reverse primer	CAT​TGA​TCT​CCG​TGA​CAG​CC
COX2	Forward primer	TGC​TGT​ACA​AGC​AGT​GGC​AA
	Reverse primer	GCA​GCC​ATT​TCC​TTC​TCT​CC
GAPDH	Forward primer	CAT​CAC​TGC​CAC​CCA​GAA​GAC​T
	Reverse primer	GAC​ACA​TTG​GGG​GTA​GGA​ACA​C

### 2.9 Inflammatory cytokines and NO detection

BV2 microglial cells were pretreated with CD for 2 h, followed by stimulation with LPS (1 μg/mL) for 24 h. Control cells were treated with DMSO. Culture supernatants were then collected and analyzed. IL-6, TNF-α and IL-1β were measured using mouse ELISA kits (IL-6, Cat:JM-02446M1, JINGMEI, Jiangsu, China; TNF-α, Cat: JM-02415M1, JINGMEI, Jiangsu, China; IL-1β, Cat:JM-02323M1, JINGMEI, Jiangsu, China) according to the manufacturer’s protocol. Absorbance was measured at 450 nm using a spectrophotometer (Thermo Scientific Multiskan Skylitch, Type 1550, USA). Besides, the concentration of total nitrite was quantified with a Griess assay kit (Cat: S0021S, Beyotime, Shanghai, China). Absorbance was measured at 540 nm using a spectrophotometer (Thermo Scientific Multiskan Skylitch, Type 1550, USA).

### 2.10 Western blot

Pretreated BV2 cells were collected, washed with PBS, and lysed at 4 °C for 40 min using RIPA buffer (Cat: P0038, Beyotime, Shanghai, China). Protein concentrations were determined using a BCA protein assay kit (Cat: P0012, Beyotime, Shanghai, China). The samples were denatured using loading buffer, separated on 10% sodium dodecyl sulfate-polyacrylamide gel electrophoresis (SDS-PAGE) gels, and subsequently transferred to Immobilon®-P PVDF membranes (Merck Millipore, catalog number IPVH00010). After blocking with 5% bovine serum albumin (BSA, Cat: A8010, Solarbio, Beijing, China) for 1 h at room temperature, the membranes were incubated with 1:1000 primary antibodies (Cell Signaling Technology, Beverly, MA, USA) against p-EGFR, EGFR, p-PI3K, PI3K, p-AKT1, AKT1, p-ERK1/2, ERK1/2, p-NF-κB p65, NF-κB p65, p-IκBα, IκBα, inducible nitric oxide synthase (iNOS), COX2, and cleaved caspase-3 and 1:5000 primary antibody against β-actin/GADPH at 4 °C overnight. Following three washes with Tris-buffered saline Tween-20 (TBST), the membranes were incubated at room temperature for 2 h with 1:5000 horseradish peroxidase (HRP)- conjugated secondary antibodies (Cell Signaling Technology, Beverly, MA, USA). After three washes with TBST to remove unbound secondary antibodies, the membranes were scanned and visualized with an enhanced chemiluminescence (ECL) substrate (Cat: BL520B, Biosharp, Beijing, China) using a chemiluminescence imaging system (Tanon, Shanghai, China). Protein abundance was quantified with ImageJ 1.53c software (National Institutes of Health, Bethesda, MD, USA).

### 2.11 Pharmacological inhibition assay

To validate the involvement of the EGFR/PI3K/AKT1/ERK/NF-κB pathway in the anti-inflammatory effect of CD, BV2 microglial cells were seeded in 6-well plates at a density of 1 × 10^5^ cells/well and cultured overnight. Cells were then pretreated with 10 μM LY294002 (PI3K inhibitor, HY-10108, MCE) or 2 μM AG-1478 (EGFR inhibitor, Cat: HY-13524, MCE) for 1 h, followed by 16 μM CD treatment for 2 h, and then stimulated with 1 μg/mL LPS for 24 h. Control cells were treated with DMSO. Cell lysates were collected and subjected to Western blot analysis to detect the phosphorylation of AKT1, NF-κB p65, and ERK1/2, as well as the protein expression of iNOS.

### 2.12 NF-κB p65 nuclear translocation detection

Pretreated BV2 cells were washed with PBS, fixed with 4% paraformaldehyde for 30 min at room temperature and then permeabilized with 0.2% Triton X-100 (Cat: T8200, Solarbio, Beijing, China) for 30 min at room temperature. After blocking with 5% BSA (BSA, Cat: A8010, Solarbio, Beijing, China) for 1 h at room temperature, the samples were incubated overnight at 4 °C with an anti-NF-κB p65 primary antibody (Cell Signaling Technology, MA, USA) diluted at 1:200. Following three washes with TBST, the samples were subsequently incubated for 2 h at room temperature with a 1:200 dilution of an Alexa Fluor® 488-labeled secondary antibody (Cat: RS23220, Immunoway, Plano, TX, USA). Cell nuclei were stained with 4′,6-diamidino-2-phenylindole (DAPI, Cat: C1006, Beyotime, Shanghai, China) for 10 min. A laser scanning confocal microscope (Olympus FV3000, Evident Corporation, Japan) was used to observe and capture images of nuclear translocation.

### 2.13 Reactive oxygen species (ROS) detection

Intracellular ROS levels in BV2 cells were measured using a Reactive Oxygen Species Assay Kit (Cat: S0033S, Beyotime, Shanghai, China). The pretreated cells were washed with PBS, and incubated with 2′,7′-dichlor ofuorescin diacetate (DCFH-DA) at 37 °C for 30 min. Nuclei were subsequently stained with DAPI for 10 min, and images were acquired through a laser scanning confocal microscope (Olympus FV3000, Evident Corporation, Japan). Fluorescence intensity was analyzed with ImageJ 1.53c software.

### 2.14 Mitochondrial membrane potential (MMP; ΔΨm) detection

Pretreated BV2 cells were stained with 5,5′,6,6′-tetrachloro-1,1′,3,3′-tetraethylbenzimadazolylc arbocyanine iodide (JC-1; Cat:C2006, Beyotime, China) for 20 min at 37 °C. Following staining, cells were washed with JC-1 staining buffer and visualized with a laser confocal microscope. JC-1 aggregates and monomers were detected at excitation/emission wavelengths of 550/590 nm and 500/529 nm, respectively. Fluorescence intensity was analyzed with ImageJ 1.53c software.

### 2.15 Apoptosis detection

SH-SY5Y cells were treated with CM for 24 h. Apoptosis was evaluated using the One- step TUNEL *In Situ* Apoptosis kit (Cat: E-CK-A321, Elabscience, Wuhan, China) according to the manufacturer’s instructions. In brief, cells were fixed with 4% paraformaldehyde for 10 min at room temperature, permeabilized with 0.2% Triton X-100 (Cat: T8200, Solarbio, Beijing, China) for 15 min and washed three times with PBS. After incubation with terminal deoxynucleotidyl transferase (TdT) Equilibration buffer at 37 °C for 10 min, cells were labeled with the mixed labeling working solution at 37 °C for 2 h. Nuclei were subsequently stained with DAPI for 10 min, and images were acquired through a laser scanning confocal microscope (Olympus FV3000, Evident Corporation, Japan). Fluorescence intensity was analyzed with ImageJ 1.53c software.

### 2.16 Statistical analysis

All data were analyzed using SPSS software (version 20.0; IBM, Armonk, NY, USA) and are presented as the mean ± standard deviation (SD) or mean ± standard error of the mean (SEM) from at least three independent biological replicates (n ≥ 3), as indicated in the figure legends. Normality of data distribution was assessed using the Shapiro-Wilk test. For comparisons between two groups, the unpaired Student’s t-test was used. For multiple-group comparisons (three or more groups), one-way analysis of variance (ANOVA) was performed, followed by Tukey’s *post hoc* test. A P value of <0.05 was considered statistically significant. All figures were generated using GraphPad Prism (version 9.0; GraphPad Software, La Jolla, CA, USA).

## 3 Results

### 3.1 Acquisition of potential targets and screening of core targets

The chemical structure of CD is presented in Figure 1A. A total of 105, 28 and 44 potential targets of CD were obtained from the Swiss Target Prediction platform, TCMSP, and BATMAN-TCM, respectively. As shown in Figure 1B, these were combined into a non-redundant set of 158 targets for subsequent analysis. From the GeneCards database, 10,781 out of 10,813 entries associated with neurodegenerative diseases were selected using a relevance score threshold greater than 1. Additionally, 147 disease-related targets were obtained from the OMIM database. Following intersection analysis, 137 overlapping targets were identified as the targets of CD.

**FIGURE 1:**
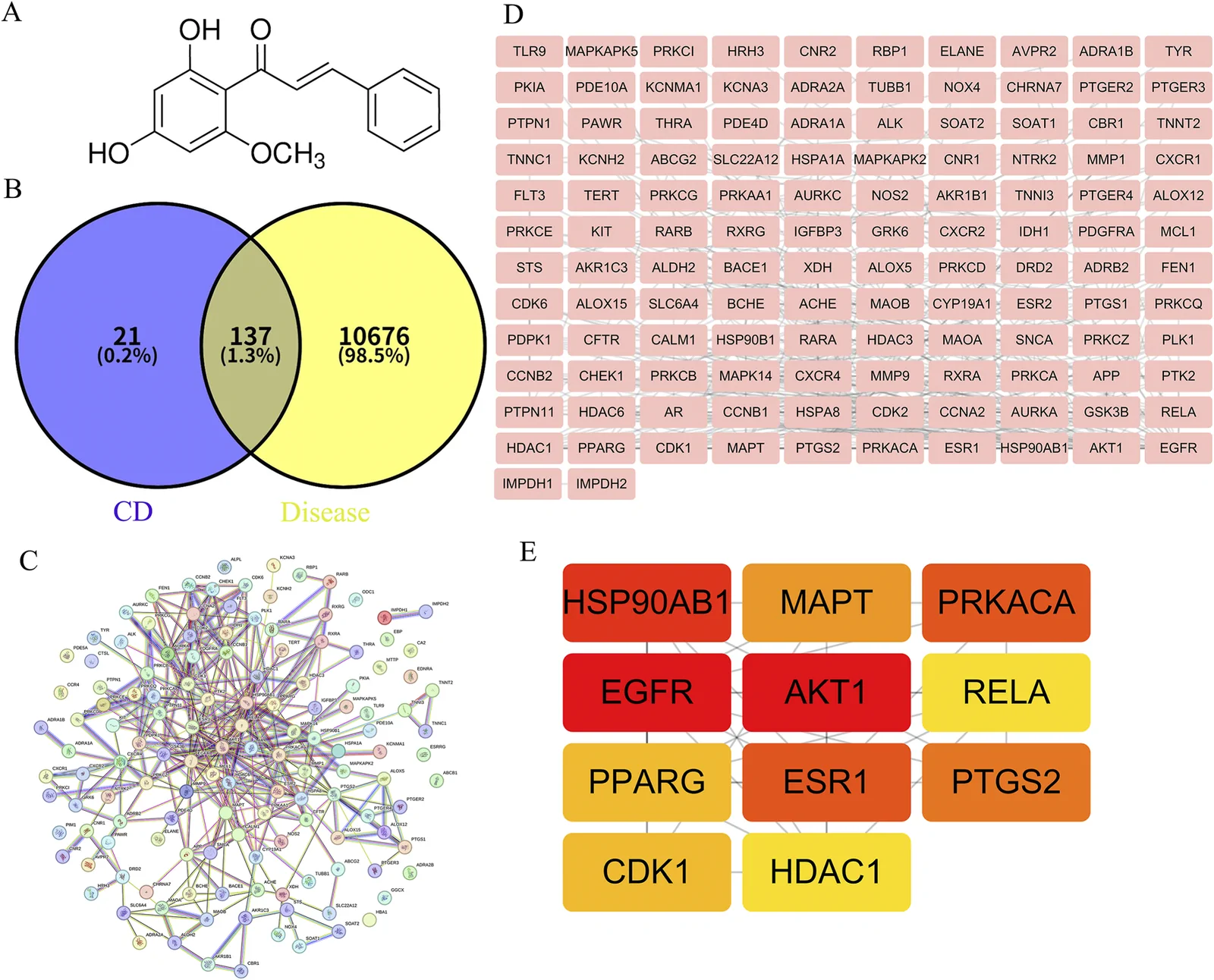
. Acquisition of potential targets and screening of core targets. **(A)** Chemical structures of CD. **(B)** The Venn. The blue circle represents predicted targets of CD, while the yellow one represents the targets related to neurodegenerative diseases. **(C)** PPI network exported by STRING database (high confidence: >0.700). **(D)** Diagram of core target construction using Cytoscape software. **(E)** PPI network built by MCODE plugin of Cytoscape.

To identify potential hub genes, a PPI network of candidate targets was constructed using the STRING database. This analysis identified 354 interaction targets at a high-confidence threshold (>0.700), as shown in Figure 1C. To further refine the core proteins in the PPI network, a topological analysis of network nodes was conducted using Cytoscape 3.9.1, leading to the identification of 122 core targets (Figure 1D). Additionally, densely connected regions within the protein interaction network were identified using the MCODE plugin in Cytoscape (Figure 1E). In this figure, the depth of the node color is proportional to the degree value. By integrating topological properties and module analysis results, 11 hub genes positioned at the core of the network were ultimately identified, including EGFR, AKT1, HSP90AB1, ESR1, PRKACA, PTGS2/(COX-2), MAPT, CDK1, PPARG, RELA, and HDAC1.

### 3.2 GO function and KEGG pathway enrichment analysis

To investigate the biological processes, subcellular localization and molecular functions involved in these core targets, GO enrichment analysis was performed. A total of 137 target genes were submitted to the DAVID database for GO analysis. GO analysis included biological process (BP), cellular component (CC), and molecular function (MF). In our study, 831 BP terms, 13 CC terms, and 102 MF terms were identified. As shown in Figure 2, the bar plot presents the enrichment fold change for BP terms (Figure 2A) and the integrated dot plot summarizes the most significant enrichment results across BP, CC, and MF categories (Figure 2B). In the BP category, the most significantly enriched terms were associated with stress response, regulation of kinase activity, and tissue development. These encompassed response to oxidative stress, positive regulation of protein localization, and peptidyl-serine phosphorylation peptidyl-serine modification- a key post-translational modification through which kinases such as EGFR and AKT1 mediate their functions. Furthermore, processes with clear tissue specificity, such as prostate glandular acinus development, were also enriched. For CC, targets were significantly enriched in plasma membrane signaling microdomains, specifically membrane rafts (also referred to as membrane microdomains), which are key sites for the activation of receptors such as EGFR and the initiation of downstream signaling. Significant enrichment was also detected in neuron-associated structures (e.g., neuronal cell body and glutamatergic synapse) and in serine/threonine protein kinase complexes. For MF, the most significantly enriched terms included ubiquitin protein ligase binding, ubiquitin-like protein ligase binding, transcription coregulator binding, RNA polymerase II-specific DNA-binding transcription factor binding, and protein serine/threonine kinase activity. Of these, protein serine/threonine kinase activity corresponds to key kinases such as AKT1, PRKACA, and CDK1. Similarly, NF-κB binding is indicative of the transcription factor RELA, and heat shock protein binding implicates HSP90AB1.

**FIGURE 2:**
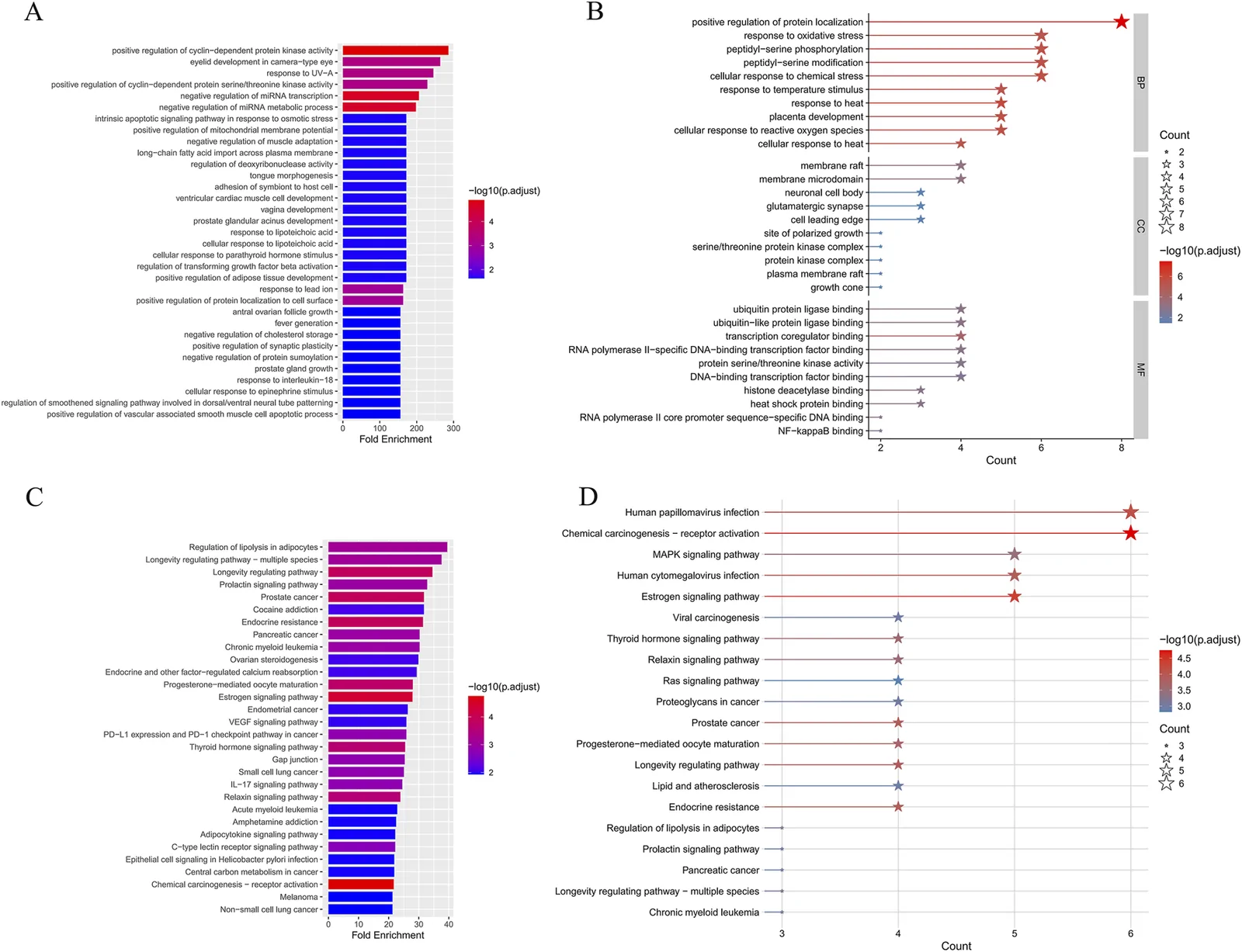
GO function and KEGG pathway enrichment analysis. **(A,B)** GO Enrichment and **(C,D)** KEGG Pathway Enrichment Analysis.

KEGG pathway enrichment analysis (*P* < 0.01) identified 116 significant pathways. As shown in Figures 2C,D, the top-ranked pathways included human papillomavirus infection, chemical carcinogen-receptor activation, MAPK signaling, and estrogen signaling. The enrichment of pathways such as human papillomavirus infection and chemical carcinogen-receptor activation largely reflects the cross-annotation of hub genes in the KEGG database. Among these, three signaling axes relevant to neuroinflammation were further analyzed: the MAPK/ERK pathway, enriched with hub genes including EGFR, AKT1, PRKACA, MAPT, and RELA; the PI3K/AKT pathway, in which EGFR and AKT1 play key roles; and the NF-κB pathway, with RELA and PTGS2 (COX-2) as core effector molecules.

### 3.3 Molecular docking analysis between CD and target proteins

As shown in Figure 3, EGFR, AKT1, HSP90AB1, IKKB, MAPK1, PTGS2, and RELA proteins were selected for molecular docking. A more negative binding energy indicates stronger binding affinity between the ligand and the protein. As shown in Table 2, the docking scores of CD with all these core targets were lower than −5 kcal/mol, suggesting potential interactions between CD and these proteins at the computational level.

**FIGURE 3:**
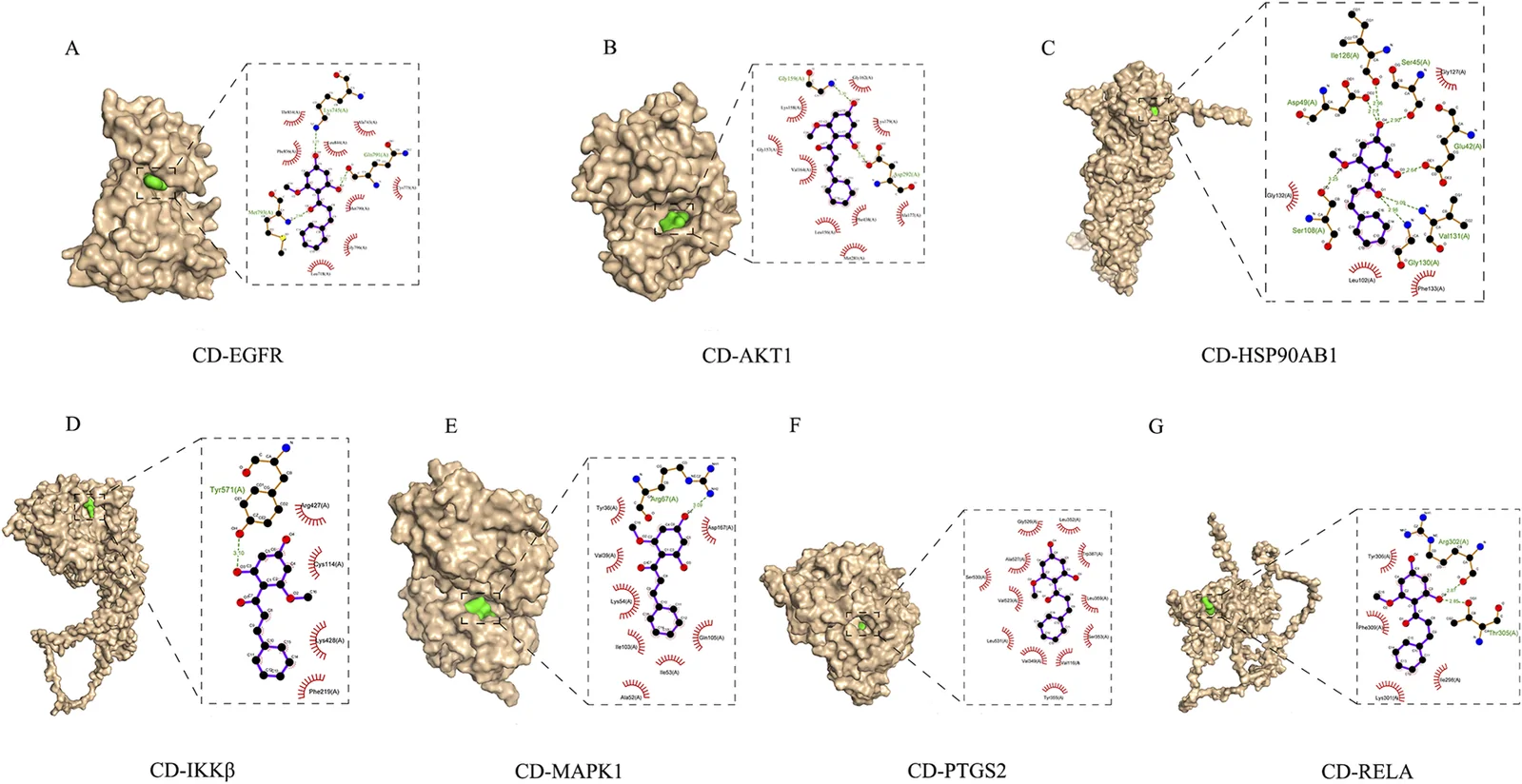
Molecular docking of CD with **(A)** EGFR, **(B) **AKT1, **(C)** HSP90AB1, **(D)** IKKβ, **(E)** MAPK1, **(F)** PTGS2, and **(G)** RELA.

**TABLE 2:** The binding energy of molecular docking.

Target protein	PDB ID/Alpha fold (AF)	Binding energy (kcal/mol)
EGFR	5ugc	−8.2
AKT1	3mvh	−8.6
HSP90AB1	7zr0	−7.7
IKKβ	AF-O14920	−7.5
MAPK1	1tvo	−7.4
PTGS2	5ikr	−7.0
RELA	AF-Q04206	−6.0

### 3.4 CD alleviated LPS-induced BV2 cell morphological changes

The effects of CD and LPS on BV2 cell viability were assessed using the CCK-8 assay. CD at concentrations ranging from 2 to 16 μM did not affect cell viability (Figure 4A). LPS at 2 and 4 μg/mL significantly reduced cell viability, indicating cytotoxic effects (Figure 4B). Based on these results, CD (2–16 μM) and LPS (1 μg/mL) were selected for subsequent experiments.

**FIGURE 4:**
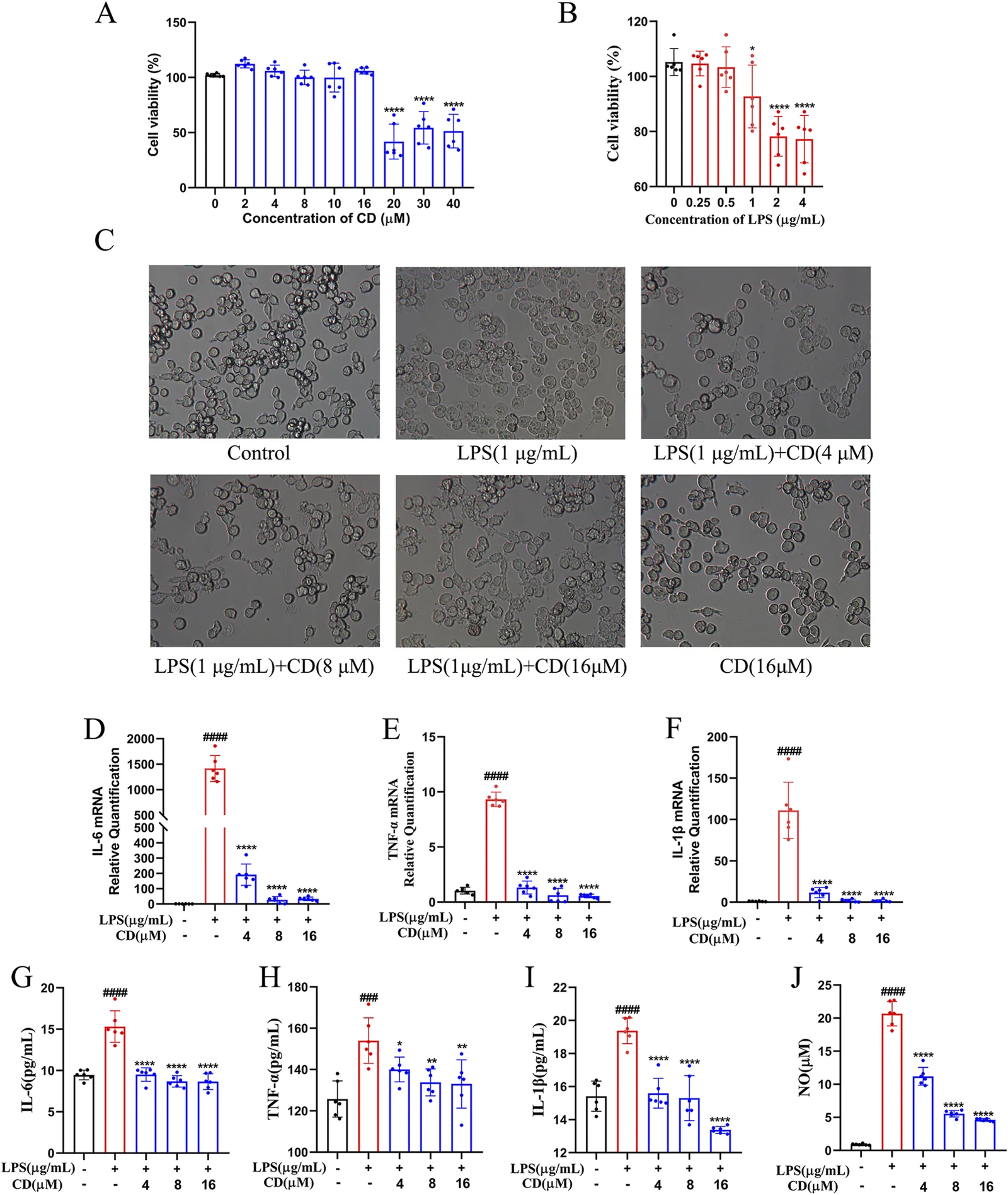
CD alleviates LPS-induced morphological changes and suppresses inflammatory responses in BV2 microglial cells. **(A)** Effect of CD on BV2 cell viability. **(B)** Effect of LPS on BV2 cell viability. Data were presented as the mean ± SD (n = 6); ****p < 0.0001, and *p < 0.05 versus the control group. **(C)** CD alleviates LPS-induced morphological changes (20X). **(D–F)** CD suppresses LPS-induced mRNA expression of IL-6, TNF-α, and IL-1β. **(G–J)** CD inhibits LPS-induced release of IL-6, TNF-α, IL-1β, and NO. Data were presented as the mean ± SD (n = 6); ^####^p < 0.0001, and ^###^p < 0.001 versus the control group; ****p < 0.0001, **p < 0.01 and *p < 0.05 versus the model group.

Next, the effect of CD on the morphological alterations in LPS-induced BV2 cells was examined. As shown in Figure 4C, cells in the control group maintained a ramified phenotype with small, slender cell bodies. As expected, 1 μg/mL of LPS induced a shift from the resting, ramified morphology to an activated, amoeboid shape, characterized by reduced branching and enlarged cell bodies (Kloss et al., 2001). Pre-treatment with CD significantly reduced the morphological alterations induced by LPS, promoting a reversion toward the ramified state.

### 3.5 CD inhibited LPS-induced mRNA expression and release of inflammatory mediators in BV2 cells

Inflammatory stimulation can induce the activation of microglia, which secrete inflammatory mediators and lead to the pathogenesis of various neurodegenerative diseases (Cai et al., 2018). To evaluate the anti-inflammatory effect of CD, RT-qPCR analysis was performed to measure the mRNA expression levels of IL-6, IL-1β, and TNF-α in BV2 cells treated with LPS. LPS stimulation markedly upregulated the expression of these pro-inflammatory cytokines. Pre-treatment with CD significantly suppressed this LPS-induced increase in IL-6, IL-1β, and TNF-α mRNA (Figures 4D–F). Furthermore, ELISA results revealed that CD also significantly reduced the LPS-induced release of IL-6, IL-1β, TNF-α, and NO into the culture supernatant (Figures 4G–J).

### 3.6 CD treatment suppressed EGFR/PI3K/AKT1 signaling pathway and ERK2 activation in LPS-induced BV2 cells

To verify the network pharmacology predictions, we investigated whether CD modulates the EGFR/PI3K/AKT1/ERK signaling pathways. BV2 Cells were pretreated with CD for 2 h, followed by stimulation with LPS for 24 h. At the transcriptional level, LPS significantly upregulated the mRNA expression of EGFR, PI3K, and AKT1, which was effectively suppressed by CD pretreatment (Figures 5A–C). At the protein level, CD similarly reversed the LPS-induced increase in the phosphorylation of EGFR, PI3K, and AKT1, as well as the activation of ERK1/2 (Figures 5D–G). Collectively, these results demonstrate that CD treatment correlates with reduced activation of the EGFR/PI3K/AKT1 and ERK signaling pathways in LPS-induced microglia.

**FIGURE 5:**
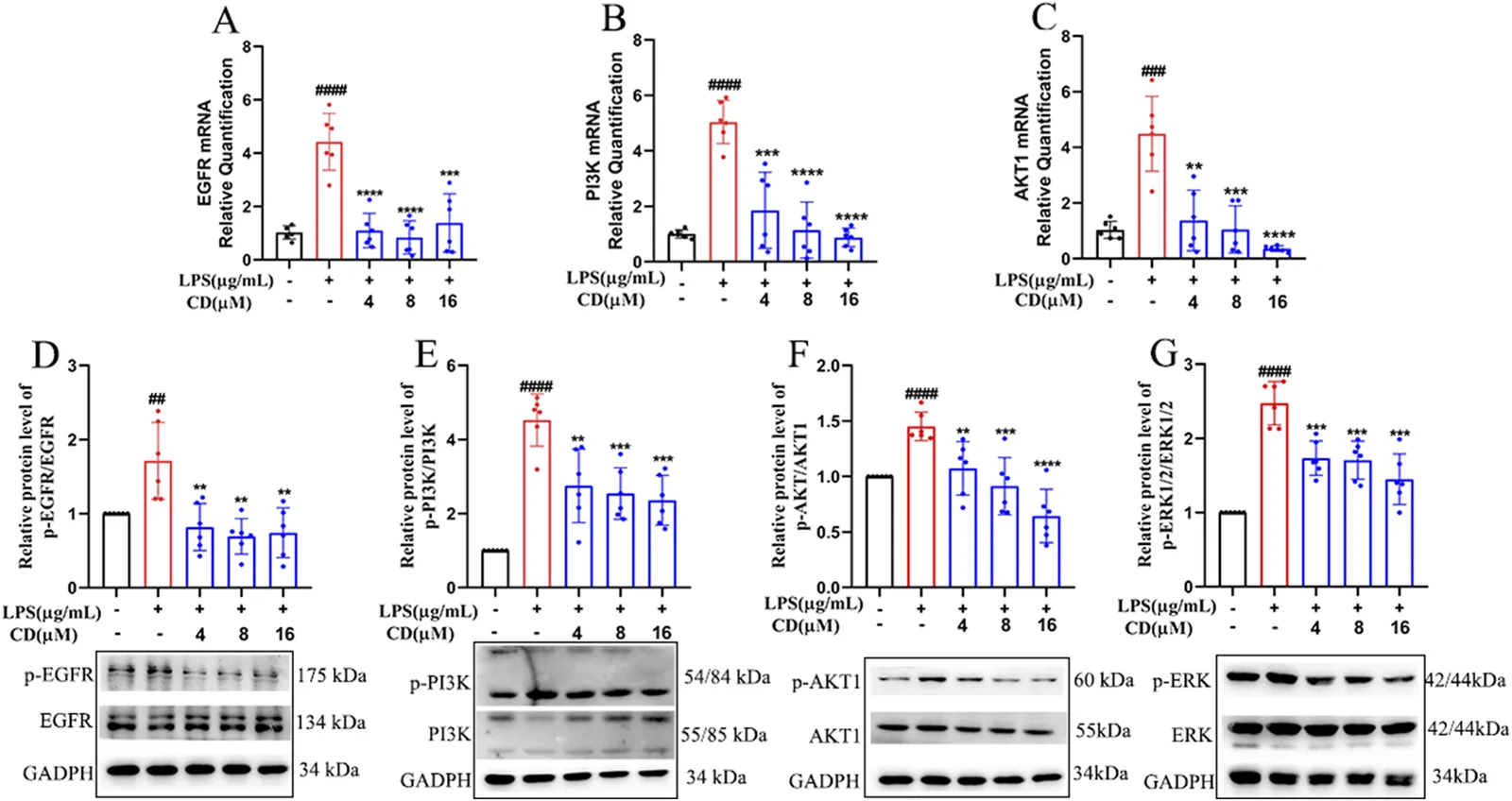
CD suppresses the EGFR/PI3K/AKT1 signaling pathway and ERK2 activation in LPS-induced BV2 cells. **(A–C)** CD downregulates LPS-induced mRNA expression of EGFR, PI3K, and AKT1 in BV2 cells. **(D–G)** CD inhibits LPS-induced phosphorylation of EGFR, PI3K, and AKT1, as well as activation of ERK1/2, at the protein level. The bars represent the mean ± SD (n = 6); ^####^p < 0.0001, ^###^p < 0.001, and ^##^p < 0.01 versus the control group; ****p < 0.0001, ***p < 0.001 and **p < 0.01 versus the model group.

### 3.7 CD treatment suppressed NF-κB signaling pathway activation in LPS-induced BV2 cells

Stimulation of the ERK and PI3K/AKT signaling pathways can activate the transcription factor NF-κB (Madrid et al., 2000; Kefaloyianni et al., 2006). We therefore assessed the inhibitory effect of CD on this pathway. qRT-PCR analysis showed that CD suppressed the LPS-induced increase in mRNA levels of NF-κBp65, IκBα, iNOS, and COX2 (Figures 6A–D). Western blot analysis indicated that CD reversed the LPS-induced phosphorylation of NF-κB p65 and IκBα, and decreased iNOS and COX-2 protein expression (Figures 6E–H). In addition, immunocytochemistry assays confirmed that CD inhibited LPS-induced nuclear translocation of NF-κB p65 (Figure 6I). Thus, these findings demonstrate that CD treatment coincides with suppression of the NF-κB signaling pathway, which is consistent with the predictions of network pharmacology analysis and molecular docking.

**FIGURE 6:**
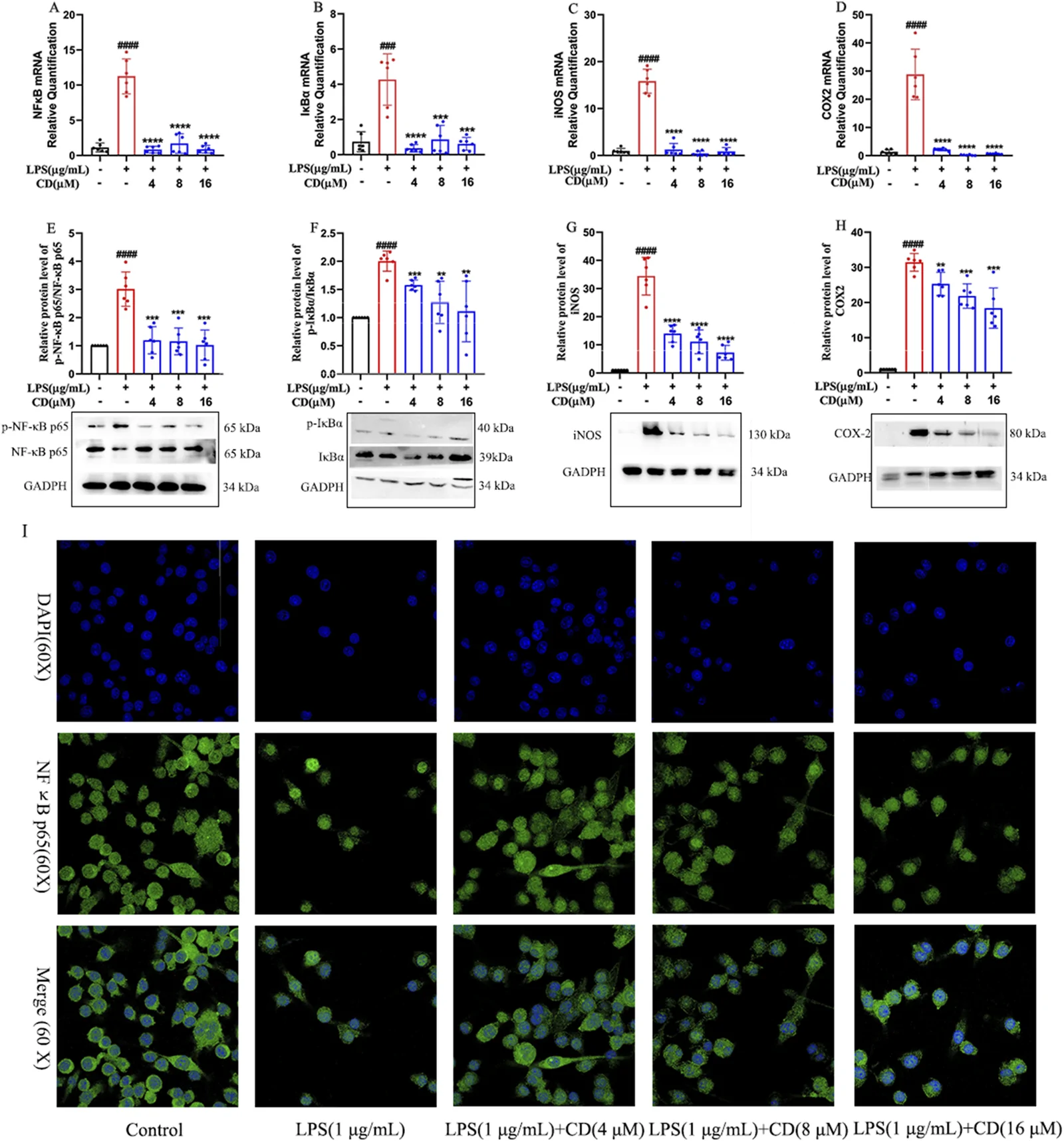
CD inhibits NF-κB signaling pathway activation in LPS-induced BV2 cells. **(A–D)** CD downregulates LPS-induced mRNA expression of NF-κBp65, IκBα, iNOS, and COX2. **(E–H)** CD suppresses LPS-induced phosphorylation of NF-κB p65 and IκBα, and reduces protein levels of iNOS and COX-2. **(I)** CD inhibits LPS-induced nuclear translocation of NF-κB p65. The bars represent the mean ± SD (n = 6); ^####^p < 0.0001, and ^###^p < 0.001 versus the control group; ****p < 0.0001, ***p < 0.001 and **p < 0.01 versus the model group.

### 3.8 CD attenuated ROS production and prevented MMP loss in LPS-induced BV2 cells

Intracellular level of ROS was measured using the DCFH-DA assay. As shown in Figures 7A,B, LPS stimulation significantly increased ROS production, as evidenced by stronger green fluorescence in the LPS-stimulated group alone compared to the control. CD pretreatment dose-dependently attenuated this LPS-induced ROS accumulation. These results indicate that CD alleviates oxidative stress in BV2 cells.

**FIGURE 7:**
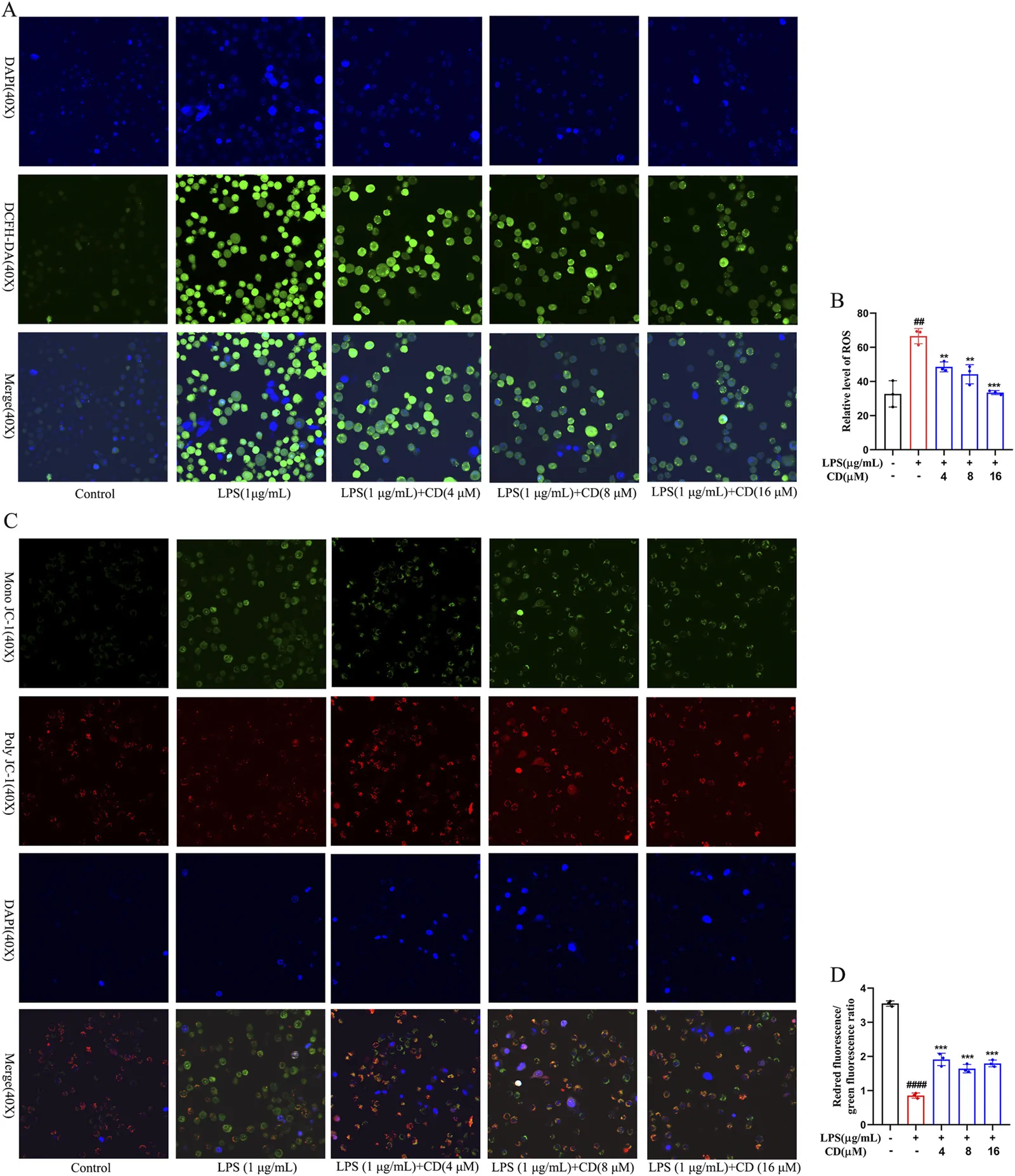
CD attenuates ROS production and prevents mitochondrial membrane potential (MMP) loss in LPS-induced BV2 cells. **(A,B)** DCFH-DA fluorescence assay showing that CD dose-dependently attenuates LPS-induced intracellular ROS accumulation in BV2 cells. **(C, D)** JC-1 fluorescence assay demonstrating that CD significantly restores LPS-induced MMP loss in BV2 cells. The bars represent the mean ± SD (n = 3); ^####^p < 0.0001, and ^##^p < 0.01 versus the control group; ***p < 0.001 and **p < 0.01 versus the model group.

Excessive ROS production damages mitochondria by inducing mitochondrial permeability transition pore (mPTP) opening and diminishing the MMP (Zhao et al., 2024). To investigate whether CD protects mitochondrial function, we measured MMP using the fluorescent probe JC-1. According to the results presented in Figures 7C,D, LPS treatment caused a collapse of MMP in BV2 cells. In contrast, pre-treatment with CD markedly prevented this LPS-induced decline in MMP.

### 3.9 CD reduced neurotoxicity in SH-SY5Y cells induced by activated microglia

Activated microglia release neurotoxic mediators that induce neuronal death upon inflammatory stimulation (Liddelow et al., 2017). To exclude the possibility that residual CD or LPS in the CM might directly affect SH-SY5Y cells, we first assessed the direct effects of CD and LPS on SH-SY5Y cells. As shown in Supplementary Figure S1, LPS alone significantly reduced SH-SY5Y cell viability and induced morphological damage, characterized by cell body shrinkage, rounding, and the retraction and degeneration of neurites, leading to a sparse and fragmented neuronal network. However, CD alone had no significant effect on SH-SY5Y cell viability or morphology. Moreover, co-treatment with CD and LPS failed to improve SH-SY5Y cell viability or morphology, indicating that CD does not exert direct protective effects on SH-SY5Y cells. Based on these results, we employed a well-established co-culture model to assess whether CD could protect SH-SY5Y neurons through modulation of microglial inflammatory responses. SH-SY5Y neuronal cells were treated for 24 h with CM collected from BV2 microglial cultures (Figure 8A). As shown in Figures 8B,C, SH-SY5Y cells treated with CM from control BV2 cells exhibited 99.2% viability and normal morphology. In contrast, CM from LPS-induced BV2 cells significantly reduced neuronal viability and impaired morphology. Importantly, CM from BV2 cells co-treated with LPS and CD significantly attenuated these detrimental effects, improving both cell viability and morphology. These results indicate that by mitigating neuroinflammatory responses in microglia, CD protects SH-SY5Y cells from microglial activation-induced neurotoxicity.

**FIGURE 8:**
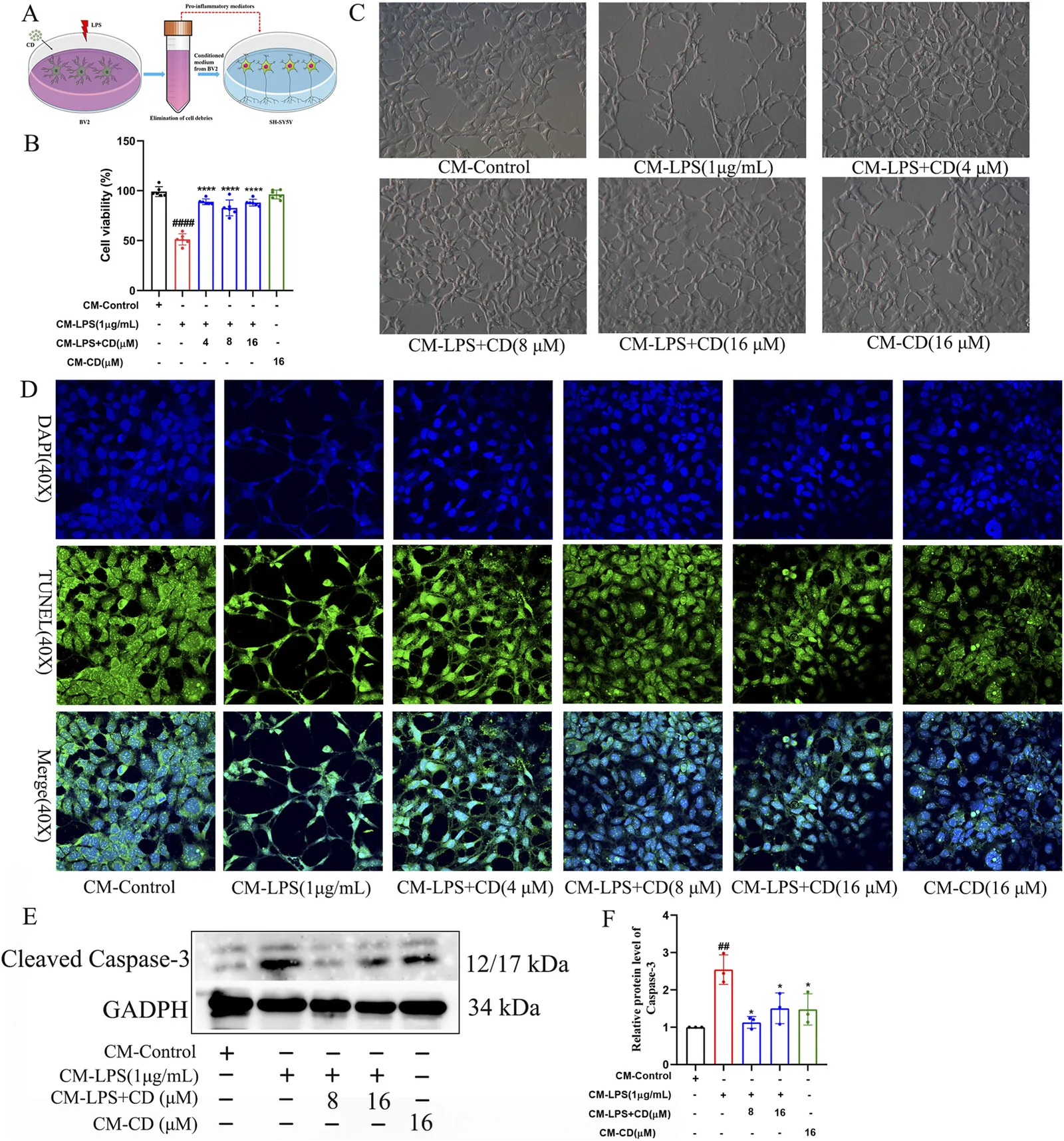
CD protects SH-SY5Y cells against microglial activation-induced neurotoxicity and apoptosis. **(A)** Schematic diagram of the effect of CM from LPS-induced BV2 microglia pretreated with CD on SH-SY5Y cells. **(B,C)** Conditioned medium (CM) from CD-pretreated BV2 cells improves viability and morphology (10X) of SH-SY5Y cells compared to CM from LPS- induced BV2 cells. Data were presented as the mean ± SD (n = 6). ####p < 0.0001 versus the CM-control group; ****p < 0.0001 versus the CM-LPS group. **(D)** TUNEL assay showing that CM from CD-pretreated BV2 cells reduces neuronal apoptosis induced by LPS-activated microglial CM. **(E,F)** CM from CD-pretreated BV2 cells inhibits the protein levels of cleaved caspase-3. The bars represent the mean ± SD (n = 3); ##p < 0.01 versus the CM-control group; *p < 0.05 versus the CM-LPS group.

### 3.10 CD reduced apoptosis in SH-SY5Y cells induced by activated microglia

CM from LPS-stimulated BV2 microglia is known to induce apoptosis in SH-SY5Y neuronal cells (Liu et al., 2020; Yang et al., 2022). To determine if CD pretreatment of microglia confers neuroprotection, we assessed apoptosis in SH-SY5Y cells using TUNEL assay after 24 h incubation with CM. As shown in Figure 8D, CM from LPS-stimulated BV2 cells significantly increased neuronal apoptosis compared to CM from control cells. This effect was markedly attenuated when BV2 cells were pretreated with CD prior to LPS stimulation. We further examined whether the observed reduction in neuronal apoptosis was associated with changes in cleaved caspase-3 expression. Western blot analysis revealed that CM from LPS-stimulated BV2 cells significantly increased cleaved caspase-3 protein expression compared to CM from control cells. This effect was also attenuated by CD pretreatment prior to LPS stimulation (Figures 8E,F). Together, these results suggest that CD may reduce neuronal apoptosis by attenuating inflammatory responses in activated microglia.

### 3.11 Pharmacological inhibition of EGFR and PI3K suppressed LPS-induced inflammatory signaling in BV2 cells

To further validate the involvement of the EGFR/PI3K/AKT1/ERK/NF-κB pathway in the anti-inflammatory effect of CD, we performed pharmacological inhibition experiments using the PI3K inhibitor LY294002 (10 μM) and the EGFR inhibitor AG1478 (2 μM) in LPS-stimulated BV2 cells. As shown in Figure 9, treatment with LY294002 or AG1478 alone significantly reduced LPS-induced phosphorylation of AKT1, NF-κB p65, and ERK1/2, as well as iNOS expression, compared with the LPS group. Notably, co-treatment of CD with either inhibitor did not produce additive or synergistic effects compared to CD or inhibitor alone, suggesting that CD may act through the EGFR/PI3K/AKT1/ERK/NF-κB signaling axis. These results provide pharmacological evidence supporting the functional involvement of this pathway in the anti-inflammatory action of CD.

**FIGURE 9:**
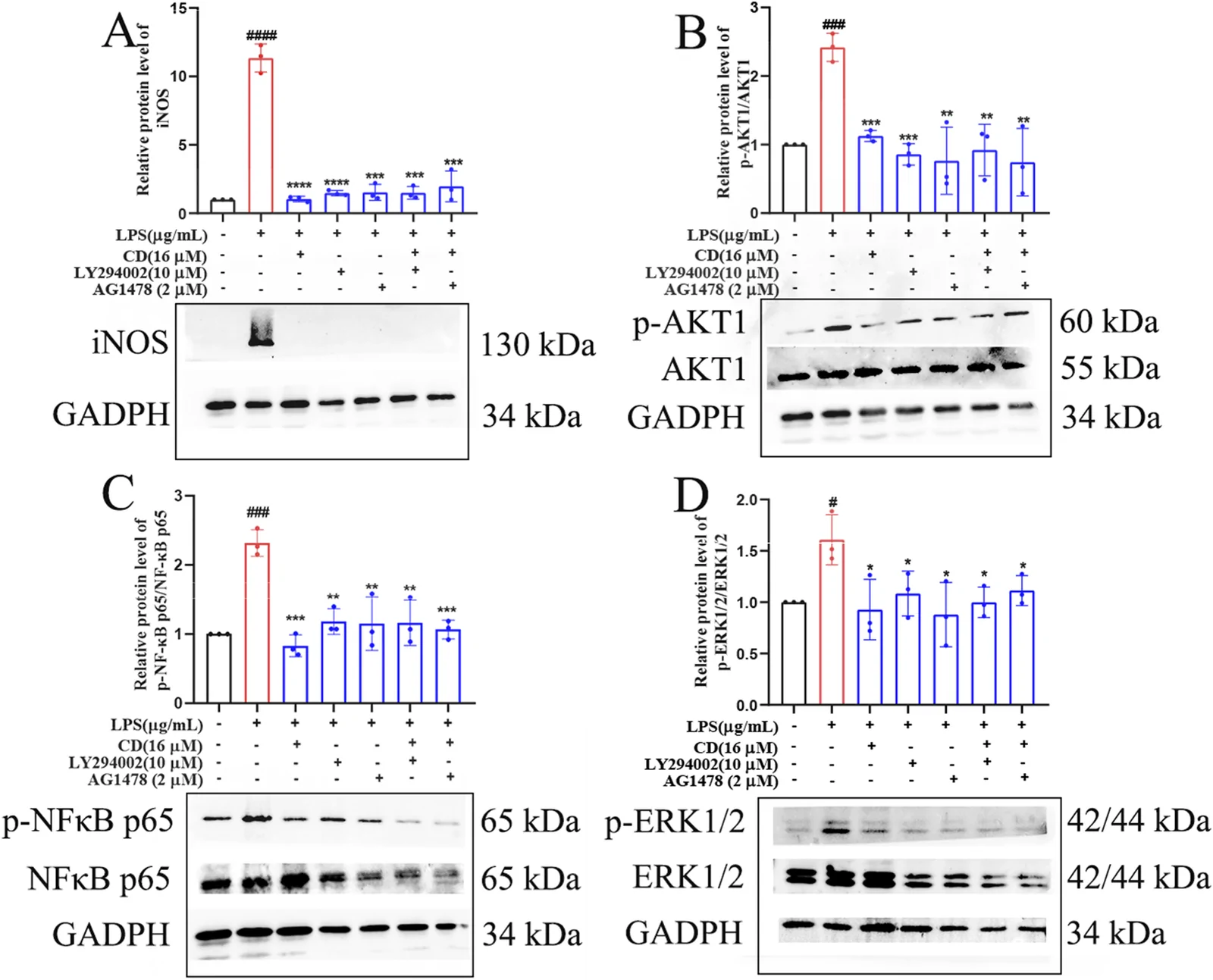
Effects of EGFR and PI3K inhibition on LPS-induced inflammatory signaling in BV2 cells. Western blot analysis of **(A)** p-AKT1/AKT1, **(B)** p-NF-κB p65/NF-κB p65, **(C)** p-ERK1/2/ERK1/2, and **(D)** iNOS expression. The bars represent the mean ± SD (n = 3); ^####^p < 0.0001, ^###^p < 0.001, and ^#^p < 0.05 versus the control group; ****p < 0.0001, ***p < 0.001, **p < 0.01, and *p < 0.05 versus the model group.

## 4 Discussion

Neuroinflammation is a key pathological driver of neurodegenerative diseases such as Alzheimer’s and Parkinson’s. Elucidating its mechanisms is therefore vital for developing therapies. This has motivated increasing interest in identifying anti-neuroinflammatory compounds from traditional medicine. In this study, we integrate network pharmacology, molecular docking, and *in vitro* experiments to systematically investigate the natural chalcone CD. Our findings suggest that CD may exert neuroprotective effects via a multi-target synergistic mechanism, providing new insights into its therapeutic potential against neuroinflammation.

Our network pharmacology and molecular docking analysis results predicted that CD may target pivotal signaling nodes, such as EGFR, AKT1, PRKACA, PTGS2 and RELA, and may regulate key pathways, including EGFR/PI3K/AKT1, MAPK/ERK, and NF-κB signaling. These predictions are consistent with previous network pharmacology studies on CD (Wu et al., 2024; Wu et al., 2025; Zhang et al., 2025). At the molecular level, CD may regulate key activities, such as kinase function, NF-κB binding, and heat shock protein interactions. This multi-target action is associated with reduced microglial overactivation, alleviated oxidative stress and mitochondrial dysfunction, and attenuated neuroinflammation, which may contribute to indirect neuroprotective effects. Among the KEGG pathways enriched in this study, some are not directly associated with neuroinflammation, such as human papillomavirus infection and chemical carcinogenesis. This phenomenon mainly reflects the inherent annotation structure of the KEGG database-core targets including EGFR, AKT1, and RELA are highly pleiotropic and serve as shared nodes across multiple signaling cascades. Therefore, while the enrichment results are broad, the present study focuses our mechanistic discussion and experimental validation on the MAPK/ERK, PI3K/AKT, and NF-κB axes, which are most relevant to microglial activation and neuroinflammation and are supported by our *in vitro* data.

PPI network analysis identified 11 core targets, including EGFR, AKT1, HSP90AB1, ESR1, PRKACA, PTGS2 (COX-2), MAPT, CDK1, PPARG, RELA, and HDAC1. Several of these targets have also been identified as core targets in previous network pharmacology studies on CD (Wu et al., 2024; Wu et al., 2025). Among them, EGFR, a member of the ERBB receptor tyrosine kinases family, plays a key role in regulating cell proliferation, differentiation, and survival (Sabbah et al., 2020). EGFR-mediated signaling has been recognized as a potential target for treating neuroinflammatory diseases (Yang et al., 2016; Huang et al., 2025). Ligand binding (e.g., through epidermal growth factor (EGF) or TNF-α) triggers EGFR phosphorylation, thereby activating the PI3K/AKT and MEK/ERK pathways, promoting the release of pro-inflammatory cytokines by microglia (Fischer et al., 2006; Li et al., 2022; Gu and Pang, 2025), enhancing migration ability, and exacerbating neuroinflammation (Qu et al., 2015). AKT1, a critical serine/threonine kinase downstream of the PI3K, regulates cell survival, metabolism, and inflammatory response (Vogiatzi and Giordano, 2007; Chen et al., 2001). Its dysregulation is closely associated with neurodegenerative diseases like Alzheimer’s and Parkinson’s (Ahmad et al., 2014; Jacquet et al., 2024). While moderate AKT1 activation supports cell survival (Stambolic and Woodgett, 2006; Siddika et al., 2022), its hyperactivation may exacerbate inflammation and oxidative stress via downstream effectors (e.g., mTOR, NF-κB) (Vergadi et al., 2017; Zenke et al., 2018), and may promote pathological protein aggregation and neuronal damage (Gnanarajan et al., 2025). Furthermore, RELA, a key NF-κB subunit, acts as a central transcriptional regulator of pro-inflammatory genes (Liu et al., 2017). PTGS2(COX-2), the inducible rate-limiting enzyme in prostaglandin synthesis, serves as a critical amplifier of neuroinflammation in glial cells (Choi et al., 2009; Woodling and Andreasson, 2016). Although the individual roles of these targets in neuroinflammation are well-established, how CD synergistically modulates multiple targets (e.g., EGFR, AKT1, RELA, PTGS2) to integrate key pathways-including EGFR/PI3K/AKT1, MAPK/ERK, and NF-κB-into a coherent anti-neuroinflammatory network remains unclear. Therefore, elucidating the multi-target synergistic mechanism of CD not only provides a novel perspective on its anti-neuroinflammatory action but also establishes atheoretical foundation for developing multi-target intervention strategies.

The GO enrichment analysis demonstrated that the potential targets of CD were significantly enriched in oxidative stress response, cellular response to chemical stress, and peptide serine phosphorylation and associated modifications, and these targets were related to molecular functions, including protein kinase activity. Notably, PPI network analysis identified core targets that perform these exact functions, including EGFR, AKT1, RELA, PTGS2, HSP90AB1 and HDAC1. AKT1 possesses intrinsic serine/threonine kinase activity (Kumar et al., 2025), and the upstream receptor EGFR is known to initiate phosphorylation-dependent signaling cascades. In particular, EGFR activates AKT1, a central component of the PI3K/AKT pathway, leading to extensive substrate phosphorylation and the transmission of stress signals into intracellular responses (Wang et al., 2000). A key role of this phosphorylation network is the control over the activity and nuclear translocation of NF-κB p65 (RELA), which subsequently promotes the expression of pro-inflammatory mediators such as COX-2 (PTGS2). This mechanism functionally connects cellular reactions to oxidative and chemical stress with the modulation of inflammatory processes (Wu et al., 2013; Lu et al., 2025). Additionally, the regulatory network is reinforced by supporting targets, including HSP90AB1, which maintains kinase protein stability, and HDAC1, a key epigenetic regulator.

KEGG pathway enrichment analysis further revealed that the potential targets of CD were significantly enriched in three interrelated signaling pathways, including the MAPK/ERK, PI3K/AKT1, and NF-κB pathways. Our results showed that multiple core targets, such as EGFR, AKT1, PRKACA, MAPT, and RELA, were enriched in the MAPK pathway. Members of the MAPK family, especially ERK1/2, play important regulatory roles in neuroinflammation. Their activation leads to the phosphorylation and activation of transcription factors such as NF-κB. Studies have shown that inhibiting ERK1/2 phosphorylation can effectively reduce the release of pro-inflammatory mediators and protect neurons from inflammation-induced damage in LPS-stimulated microglia-neuron co-culture systems (Temviriyanukul et al., 2022). Sustained ERK1/2 phosphorylation has been suggested as a key driver of chronic neuroinflammation mediated by microglia and promotes progressive neurodegeneration (Chen et al., 2022). Furthermore, a preclinical study on Parkinson’s disease demonstrated that dual inhibition of ERK1/2 and NF-κB p65 phosphorylation produced a synergistic anti-inflammatory and neuroprotective effect (Chen et al., 2018). Concurrently, the PI3K/AKT1 signaling pathway represents another important mechanism associated with the neuroprotective effects of CD against neuroinflammation, with EGFR and AKT1 acting as key components across multiple enriched pathways (He et al., 2021; Liu et al., 2022). Previous studies have confirmed that the PI3K/Akt pathway is required for LPS-induced microglial activation, and that inhibition of the PI3K/Akt signaling pathway significantly suppresses the production of pro-inflammatory cytokines (e.g., IL-1β, IL-6, TNF-α) (Cianciulli et al., 2016; Saponaro et al., 2012). Furthermore, PI3K/Akt phosphorylation modulates the NF-κB signaling cascade by influencing the nuclear translocation and transcriptional activity of NF-κB p65. These signaling cascades ultimately converge on the NF-κB pathway, which functions as a master regulator of inflammatory gene expression (Saponaro et al., 2012; Dilshara et al., 2014). Moreover, in our study, RELA (a key subunit of NF-κB) and PTGS2 (COX-2) act as key effector molecules, and these targets are embedded in a highly interconnected pathway network. Based on the above research results, we propose that the anti-inflammatory effect of CD may involve its action on targets such as EGFR and coincides with regulation of the PI3K/AKT1 and MAPK/ERK signaling pathways. These signals may converge on the activation of NF-κB p65, which could subsequently modulate the transcriptional expression of inflammatory mediators such as PTGS2, thus providing a molecular basis for the anti-inflammatory action of CD. Furthermore, molecular docking results predicted that CD may interact with EGFR, AKT1, HSP90AB1, IKKβ, ERK2, COX-2, and NF-κB p65 with favorable (more negative) binding energies, suggesting potential interactions at the computational level.

To validate the results predicted by bioinformatics, we first conducted *in vitro* experiments using LPS-induced BV2 microglial cells. Microglia are resident immune cells in the central nervous system and play a key role in neuroinflammation. Upon activation, they release a large amount of pro-inflammatory cytokines, such as TNF-α, IL-6, and IL-1β, and significantly upregulate iNOS, leading to excessive production of NO. At the same time, COX-2, which is rapidly induced during inflammation, helps synthesize inflammatory mediators such as prostaglandins, thereby further amplifying the inflammatory cascade (Block and Hong, 2005). These reactions collectively promote neurodegenerative processes, leading to neuronal loss and exacerbating brain damage (Stephenson et al., 2018). Our research results showed that CD at concentrations ranging from 2 to 16 μM did not affect cell viability, and CD (4, 8, and 16 µM) significantly inhibited the expression of IL-1β, IL-6, and TNF-α in LPS-induced BV2 cells, reduced NO release, and decreased the expression of iNOS and COX-2 at both mRNA and protein levels. These results are consistent with previously reported anti-inflammatory effects of CD in LPS-induced BV2 microglial cells, with effective concentrations ranging from 6.25 to 50 μM (Chow et al., 2012). Although pharmacokinetic studies have shown that the oral bioavailability of CD is limited, and the exposure level in plasma after oral administration is lower than the effective concentration used in in *vitro* experiments (Jaiswal et al., 2017; Jaiswal et al., 2015). *In vivo* studies have demonstrated that CD at doses of 5–20 mg/kg improves cognitive function, reduces Aβ levels in the frontal cortex and hippocampus, and significantly decreases neuroinflammatory markers including IL-1β, IL-6, and TNF-α in 5XFAD transgenic mice (Smail, 2024). Similarly, in a rat model of sciatic nerve crush injury, CD at doses of 5, 10, and 20 mg/kg promoted motor and sensory recovery through anti-inflammatory, neurotrophic, and anti-apoptotic effects (Hasan et al., 2025). In a mouse model of cerebral ischemia/reperfusion injury, CD attenuated brain injury by activating the HIF-1α/VEGFA pathway (Ni et al., 2022). These findings suggest that the *in vivo* neuroprotective effects of CD may not rely on high-concentration direct exposure to the central nervous system, but rather functions through indirect mechanisms such as regulating peripheral immune signals. We further investigated the mechanism by which CD exerts neuroprotective effects against neuroinflammation at the signaling pathway level. The experimental results showed that CD significantly reduced the mRNA expression of LPS-induced EGFR, PI3K, and AKT1, and inhibited their protein phosphorylation. Consistently, CD pretreatment decreased the mRNA levels of NF-κB p65 and IκBα, attenuated the phosphorylation of NF-κB p65, IκBα, and ERK1/2 proteins, and inhibited the nuclear translocation of NF-κB p65. Mechanistically, the increase in NF-κB p65 total mRNA following LPS stimulation might be explained by a positive feedback mechanism, whereby nuclear-localized NF-κB promotes transcription of its own subunits, including p65. The elevated IκBα mRNA, in turn, could be related to the classical negative feedback loop, as IκBα is a direct transcriptional target of NF-κB (Sun et al., 1993). For the increase in the ratio of p-p65/p65 and p-IκBα/IκBα after LPS stimulation, one possible explanation is that LPS activates the IKK complex, leading to IKKβ-mediated phosphorylation of IκBα, which targets IκBα for ubiquitination and degradation, thereby releasing p65 for nuclear translocation and target gene transcription (Bhh and Jass, 2013). Thus, the increase in these ratios may reflect IKK-mediated phosphorylation events rather than an increase in total protein synthesis. In our CD-treated groups, CD suppressed the LPS-induced increases in p-p65/p65 and p-IκBα/IκBα ratios, while also reducing p65 and IκBα mRNA levels, suggesting that CD may affect both phosphorylation activation and downstream transcriptional feedback. In addition, to further validate the functional involvement of this pathway, we performed pharmacological inhibition experiments using the PI3K inhibitor LY294002 and the EGFR inhibitor AG-1478 in LPS-stimulated BV2 cells. The results showed that pretreatment with LY294002 or AG-1478 alone mimicked the inhibitory effects of CD on the above signaling pathways, and no additive effects were observed when CD was combined with either inhibitor. These findings suggest that CD exerts its anti-neuroinflammatory effects by suppressing the activation of EGFR/PI3K/AKT1/ERK1/2 signal pathway. This leads to a reduction in the activity of downstream transcription factors such as NF-κB, ultimately resulting in decreased production of inflammatory mediators (e.g., IL-1β, IL-6, TNF-α, NO) and key inflammatory enzymes (iNOS, COX-2).

Given that GO analysis indicated that the potential targets of CD were significantly enriched in the biological process “response to oxidative stress”, we further investigated its protective effect on oxidative damage in BV2 cells. Studies have shown that excessive activation of microglia not only initiates inflammatory responses but also promotes oxidative stress. This leads to an increase in ROS production, which in turn causes damage to mitochondrial structure and function as well as a decrease in MMP (Li et al., 2021; Chuang et al., 2013). Our results demonstrated that CD significantly reduced the ROS levels in BV2 cells and effectively attenuated the LPS-induced decrease in MMP.

Studies have shown that the interaction between activated microglia and neurons is involved in the pathogenesis of various neurodegenerative diseases (Shie et al., 2011). Activated microglia secrete excessive inflammatory mediators, triggering a series of reactions that leads to neuronal damage or death (Liu and Hong, 2003; Flury et al., 2025). To model the neurotoxic effects induced by activated microglia through the release of inflammatory factors, we employed a co-culture system using CM from BV2 microglia and SH-SY5Y neuronal cells. The BV2 (mouse microglial) and SH-SY5Y (human neuronal) cell lines are frequently used together in neuroinflammation research as a model to study microglia-neuron interactions mediated by inflammation in the context of neurodegenerative diseases (Qin et al., 2024; Zhu et al., 2020). Using this system, we investigated whether CD could attenuate neurotoxic damage in SH-SY5Y cells induced by microglial activation. To exclude the possibility that residual CD in the conditioned medium might directly affect SH-SY5Y cells, we first verified that CD alone had no direct protective effect on SH-SY5Y cell viability or morphology (Supplementary Figure S1). CM from LPS-stimulated BV2 cells induced neurotoxicity, apoptosis and increased the expression of cleaved caspase-3 in SH-SY5Y cells, which was consistent with previous reports (Zhu et al., 2020; Yang et al., 2017). Notably, pretreatment of BV2 cells with CD (4, 8 and 16 μM) suppressed the release of inflammatory mediators, and CM significantly alleviated neurotoxicity, neuronal apoptosis and the expression of cleaved caspase-3 in SH-SY5Y cultures. Together, these results suggest that CD may reduce neuronal apoptosis by attenuating inflammatory responses in activated microglia.

Although our study shows some advantages over previous ones, several limitations should be acknowledged. First, our findings are primarily based on BV2 and SH-SY5Y immortalized cell lines, and validation in primary microglia and primary neurons is lacking. In addition, the cross-species BV2/SH-SY5Y conditioned medium model may not fully recapitulate the complexity of syngeneic microglia-neuron interactions or the three-dimensional tissue microenvironment *in vivo*. Furthermore, the *in vivo* anti-neuroinflammatory efficacy of CD remains to be confirmed in animal models.

## 5 Conclusion

In conclusion, the present study suggests that CD alleviates oxidative stress, restores mitochondrial membrane potential, and reduces inflammatory responses in LPS-stimulated BV2 microglia, and these effects are associated with the modulation of the EGFR/PI3K/AKT1/ERK/NF-κB signaling pathway. Additionally, CD reduces neuronal apoptosis by attenuating inflammatory responses in activated microglia. These findings provide experimental evidence supporting CD as a potential therapeutic agent for the treatment of neuroinflammation-associated neurodegenerative disorders.

## Data Availability

The raw data supporting the conclusions of this article will be made available by the authors, without undue reservation.

## Glossary

Aβ: β-amyloid
AKT1: Rac-alpha serine/threonine-protein kinase
BP: Biological process
BSA: Bovine serum albumin
CC: Cellular component
CD: Cardamonin, 2′,4′-dihydroxy-6′-methoxychalcone
CDK1: Cyclin-dependent kinase 1
CM: Conditioned medium
DAPI: 4′,6-diamidino-2-phenylindole
DAVID: Database for Annotation Visualization and Integrated Discovery
DCFH-DA: 2′,7′ -dichlorofuorescin diacetate
DMEM: Dulbecco’s Modified Eagle Medium
EGF: Epidermal growth factor
EGFR: Epidermal growth factor receptor
ESR1: Estrogen receptor 1
FBS: Fetal bovine serum
FDR: False discovery rate
GAPDH: Glyceraldehyde 3-phosphate de hydrogenase
GO: Gene Ontology
HDAC1: Histone deacetylase 1
HSP90AB1: Heat shock protein 90 alpha family class B member 1
IFN-γ: Interferon-γ
IL-1β: Interleukin-1β
IL-6: Interleukin-6
iNOS: Inducible nitric oxide synthase
KEGG: Kyoto Encyclopedia of Genes and Genomes
LPS: Lipopolysaccharide
MAPT: Microtubule-associated protein tau
MF: Molecular function
MMP: Mitochondrial Membrane Potential
NO: Nitric oxide
PPARG: Peroxisome proliferator-activated receptor gamma
PPI: Protein-protein interaction
PRKACA: Protein kinase A catalytic subunit alpha
PTGS2/COX-2: Prostaglandin-endoperoxide synthase 2
RELA: Transcription factor p65
ROS: Reactive oxygen species
PCR RT-qPCR: Real-time quantitative
SDS-PAGE: Sodium dodecyl sulfate-polyacrylamide gel electrophoresis
TBST: Tris-buffered saline Tween-20
TCMSP: Traditional Chinese Medicine Systems Pharmacology Database and Analysis Platform
TNF-α: Tumor necrosis factor-α

## References

[B1] Ahmad F. Nidadavolu P. Durgadoss L. Ravindranath V. (2014). Critical cysteines in Akt1 regulate its activity and proteasomal degradation: implications for neurodegenerative diseases. Free Radical Biology and Medicine 74, 118–128. 10.1016/j.freeradbiomed.2014.06.004 24933620

[B2] Barber K. Mendonca P. Evans J. A. Soliman K. F. A. (2023). Antioxidant and anti-inflammatory mechanisms of cardamonin through Nrf2 activation and NF-kB suppression in LPS-activated BV-2 microglial cells. Int. Journal Molecular Sciences 24 (13), 10872. 10.3390/ijms241310872 PMC10341801 37446045

[B3] Bhh B. Jass J. A. (2013). The complexity of NF-κB signaling in inflammation and cancer. Mol. Cancer, 86. 10.1186/1476-4598-12-86 PMC3750319 23915189

[B4] Block M. L. Hong J. S. (2005). Microglia and inflammation-mediated neurodegeneration: multiple triggers with a common mechanism. Prog. Neurobiology 76 (2), 77–98. 10.1016/j.pneurobio.2005.06.004 16081203

[B5] Cai B. Seong K. J. Bae S. W. Chun C. Kim W. J. Jung J. Y. (2018). A synthetic diosgenin primary amine derivative attenuates LPS-stimulated inflammation *via* inhibition of NF-κB and JNK MAPK signaling in microglial BV2 cells. Int. Immunopharmacology 61, 204–214. 10.1016/j.intimp.2018.05.021 29890414

[B6] Chen W. S. Xu P. Z. Gottlob K. Chen M. L. Sokol K. Shiyanova T. (2001). Growth retardation and increased apoptosis in mice with homozygous disruption of the Akt1 gene. Genes and Development 15 (17), 2203–2208. 10.1101/gad.913901 11544177 PMC312770

[B7] Chen G. Liu J. Jiang L. Ran X. He D. Li Y. (2018). Peiminine protects dopaminergic neurons from inflammation-induced cell death by inhibiting the ERK1/2 and NF-κB signalling pathways. Int. Journal Molecular Sciences 19 (3), 821. 10.3390/ijms19030821 29534526 PMC5877682

[B8] Chen S. H. Han S. Hu C. F. Zhou R. Gao Y. Tu D. (2022). Activation of the MAC1-ERK1/2-NOX2 pathway is required for LPS-induced sustaining reactive microgliosis, chronic neuroinflammation and neurodegeneration. Antioxidants Basel, Switz. 11 (6), 1202. 10.3390/antiox11061202 PMC9220294 35740099

[B9] Choi S. H. Aid S. Bosetti F. (2009). The distinct roles of cyclooxygenase-1 and -2 in neuroinflammation: implications for translational research. Trends Pharmacological Sciences 30 (4), 174–181. 10.1016/j.tips.2009.01.002 19269697 PMC3379810

[B10] Chow Y. L. Lee K. H. Vidyadaran S. Lajis N. H. Akhtar M. N. Israf D. A. (2012). Cardamonin from Alpinia rafflesiana inhibits inflammatory responses in IFN-γ/LPS-stimulated BV2 microglia *via* NF-κB signalling pathway. Int. Immunopharmacology 12 (4), 657–665. 10.1016/j.intimp.2012.01.009 22306767

[B11] Chuang D. Y. Chan M. H. Zong Y. Sheng W. He Y. Jiang J. H. (2013). Magnolia polyphenols attenuate oxidative and inflammatory responses in neurons and microglial cells. J. Neuroinflammation 10, 15. 10.1186/1742-2094-10-15 23356518 PMC3576246

[B12] Cianciulli A. Calvello R. Porro C. Trotta T. Salvatore R. Panaro M. A. (2016). PI3k/Akt signalling pathway plays a crucial role in the anti-inflammatory effects of curcumin in LPS-activated microglia. Int. Immunopharmacology 36, 282–290. 10.1016/j.intimp.2016.05.007 27208432

[B13] Dai B. Ding X. Zhang H. Huang S. Ye L. Chen R. (2025). Garcinol promotes recovery after spinal cord injury in mouse by modulating microglial polarization *via* EGFR/PI3K/AKT signaling. Spine. 51 (6), E154–E166. 10.1097/BRS.0000000000005588 41380138

[B14] Daina A. Michielin O. Zoete V. (2019). SwissTargetPrediction: updated data and new features for efficient prediction of protein targets of small molecules. Nucleic Acids Research 47 (W1), W357–W364. 10.1093/nar/gkz382 31106366 PMC6602486

[B15] Dilshara M. G. Lee K. T. Jayasooriya R. G. Kang C. H. Park S. R. Choi Y. H. (2014). Downregulation of NO and PGE2 in LPS-stimulated BV2 microglial cells by trans-isoferulic acid *via* suppression of PI3K/Akt-dependent NF-κB and activation of Nrf2-mediated HO-1. Int. Immunopharmacology 18 (1), 203–211. 10.1016/j.intimp.2013.11.020 24291391

[B16] Fakhri S. Iranpanah A. Gravandi M. M. Moradi S. Z. Ranjbari M. Majnooni M. B. (2021). Natural products attenuate PI3K/Akt/mTOR signaling pathway: a promising strategy in regulating neurodegeneration. Phytomedicine International Journal Phytotherapy Phytopharmacology 91, 153664. 10.1016/j.phymed.2021.153664 34391082

[B17] Fischer O. M. Hart S. Ullrich A. (2006). Dissecting the epidermal growth factor receptor signal transactivation pathway. Methods Molecular Biology Clift. NJ 327, 85–97. 10.1385/1-59745-012-X:85 16780214

[B18] Flury A. Aljayousi L. Park H. J. Khakpour M. Mechler J. Aziz S. (2025). A neurodegenerative cellular stress response linked to dark microglia and toxic lipid secretion. Neuron 113 (4), 554–571.e14. 10.1016/j.neuron.2024.11.018 PMC12481204 39719704

[B19] Gnanarajan R. Uvarajan D. Ravichandran N. Karuppusamy P. A. Krishnamoorthy K. Vellingiri B. (2025). Agnuside enhances neuronal survival and cognitive function by modulating PI3K/AKT/mTOR signaling in aluminium chloride induced Alzheimer's disease. Prog. Neuro-Psychopharmacology and Biological Psychiatry 142, 111532. 10.1016/j.pnpbp.2025.111532 41120087

[B20] Gonçalves L. M. Valente I. M. Rodrigues J. A. (2014). An overview on cardamonin. J. Medicinal Food 17 (6), 633–640. 10.1089/jmf.2013.0061 24433078 PMC4060836

[B21] Gu M. Pang Z. (2025). Luteolin inhibits inflammation and M1 macrophage polarization in the treatment of Pseudomonas aeruginosa-induced acute pneumonia through suppressing EGFR/PI3K/AKT/NF-κB and EGFR/ERK/AP-1 signaling pathways. Phytomedicine International Journal Phytotherapy Phytopharmacology 141, 156663. 10.1016/j.phymed.2025.156663 40133026

[B22] Hasan W. B. Kakoo A. Kheder A. H. Smail S. W. Abdolmaleki A. Ghayour M. B. (2025). Neuroregenerative effects of cardamonin in a rat model of sciatic nerve crush injury. Neurological Research, 1–17. 10.1080/01616412.2025.2591181 41289366

[B23] He Y. Sun M. M. Zhang G. G. Yang J. Chen K. S. Xu W. W. (2021). Targeting PI3K/Akt signal transduction for cancer therapy. Signal Transduction Targeted Therapy 6 (1), 425. 10.1038/s41392-021-00828-5 PMC8677728 34916492

[B24] Huang H. J. Tseng Y. J. Lee I. J. Lo Y. L. Lin A. M. (2025). Involvement of EGFR-AKT signaling in hemin-induced neurotoxicity. Exp. Biology Medicine (Maywood, NJ) 250, 10554. 10.3389/ebm.2025.10554 PMC12121490 40444141

[B25] Jacquet R. G. González Ibáñez F. Picard K. Funes L. Khakpour M. Gouras G. K. (2024). Microglia degrade Alzheimer's amyloid-beta deposits extracellularly *via* digestive exophagy. Cell Reports 43 (12), 115052. 10.1016/j.celrep.2024.115052 PMC11760508 39644493

[B26] Jaiswal S. Sharma A. Shukla M. Lal J. (2015). Gender-related pharmacokinetics and bioavailability of a novel anticancer chalcone, cardamonin, in rats determined by liquid chromatography tandem mass spectrometry. J. Chromatography B, Anal. Technologies Biomedical Life Sciences 986-987, 23–30. 10.1016/j.jchromb.2015.01.041 25703949

[B27] Jaiswal S. Shukla M. Sharma A. Rangaraj N. Vaghasiya K. Malik M. Y. (2017). Preclinical pharmacokinetics and ADME characterization of a novel anticancer chalcone, cardamonin. Drug Testing Analysis 9 (8), 1124–1136. 10.1002/dta.2128 27794181

[B28] Kefaloyianni E. Gaitanaki C. Beis I. (2006). ERK1/2 and p38-MAPK signalling pathways, through MSK1, are involved in NF-kappaB transactivation during oxidative stress in skeletal myoblasts. Cell. Signalling 18 (12), 2238–2251. 10.1016/j.cellsig.2006.05.004 16806820

[B29] Kloss C. U. Bohatschek M. Kreutzberg G. W. Raivich G. (2001). Effect of lipopolysaccharide on the morphology and integrin immunoreactivity of ramified microglia in the mouse brain and in cell culture. Exp. Neurology 168 (1), 32–46. 10.1006/exnr.2000.7575 11170719

[B30] Kumar B. H. Kabekkodu S. P. Pai K. S. R. (2025). Structural insights of AKT and its activation mechanism for drug development. Mol. Diversity 29 (6), 5443–5463. 10.1007/s11030-025-11132-7 PMC12638386 40009150

[B31] Li W. Wang S. Zhang H. Li B. Xu L. Li Y. (2021). Honokiol restores microglial phagocytosis by reversing metabolic reprogramming. J. Alzheimer's Disease JAD 82 (4), 1475–1485. 10.3233/jad-210177 34151796

[B32] Li Q. Li Z. Luo T. Shi H. (2022). Targeting the PI3K/AKT/mTOR and RAF/MEK/ERK pathways for cancer therapy. Mol. Biomedicine 3 (1), 47. 10.1186/s43556-022-00110-2 PMC9768098 36539659

[B33] Liddelow S. A. Guttenplan K. A. Clarke L. E. Bennett F. C. Bohlen C. J. Schirmer L. (2017). Neurotoxic reactive astrocytes are induced by activated microglia. Nature 541 (7638), 481–487. 10.1038/nature21029 28099414 PMC5404890

[B34] Liu B. Hong J. S. (2003). Role of microglia in inflammation-mediated neurodegenerative diseases: mechanisms and strategies for therapeutic intervention. J. Pharmacology Experimental Therapeutics 304 (1), 1–7. 10.1124/jpet.102.035048 12490568

[B35] Liu Z. Guo F. Wang Y. Li C. Zhang X. Li H. (2016). BATMAN-TCM: a bioinformatics analysis tool for molecular mechANism of traditional Chinese medicine. Sci. Reports 6, 21146. 10.1038/srep21146 26879404 PMC4754750

[B36] Liu T. Zhang L. Joo D. Sun S. C. (2017). NF-κB signaling in inflammation. Signal Transduction Targeted Therapy 2, 17023. 10.1038/sigtrans.2017.23 29158945 PMC5661633

[B37] Liu Y. Fu Y. Zhang Y. Liu F. Rose G. M. He X. (2020). Butein attenuates the cytotoxic effects of LPS-stimulated microglia on the SH-SY5Y neuronal cell line. Eur. Journal Pharmacology 868, 172858. 10.1016/j.ejphar.2019.172858 31837307

[B38] Liu C. Gao Z. W. Wang X. Lin F. Zhang H. Z. Dong K. (2022). CD73 promotes cervical cancer growth *via* EGFR/AKT1 pathway. Transl. Cancer Research 11 (5), 1089–1098. 10.21037/tcr-21-2446 PMC9189233 35706815

[B39] Lu Y. Zhou R. Zhu R. Wu X. Liu J. Ma Y. (2025). Baicalin ameliorates neuroinflammation by targeting TLR4/MD2 complex on microglia *via* PI3K/AKT/NF-κB signaling pathway. Neuropharmacology 267, 110296. 10.1016/j.neuropharm.2025.110296 39798687

[B40] Lv S. Chen Q. Li Z. Zhou Z. (2021). An evidence update on the protective mechanism of tangeretin against neuroinflammation based on network pharmacology prediction and transcriptomic analysis. Eur. Journal Pharmacology 906, 174094. 10.1016/j.ejphar.2021.174094 34087222

[B41] Madrid L. V. Wang C. Y. Guttridge D. C. Schottelius A. J. Baldwin A. S. Jr. Mayo M. W. (2000). Akt suppresses apoptosis by stimulating the transactivation potential of the RelA/p65 subunit of NF-kappaB. Mol. Cellular Biology 20 (5), 1626–1638. 10.1128/mcb.20.5.1626-1638.2000 10669740 PMC85346

[B42] Moise G. Jîjie A. R. Moacă E. A. Predescu I. A. Dehelean C. A. Hegheş A. (2024). Plants' impact on the human brain-exploring the neuroprotective and neurotoxic potential of plants. Pharm. Basel, Switz. 17 (10), 1339. 10.3390/ph17101339 PMC11510325 39458980

[B43] Nakajima K. Kohsaka S. (2004). Microglia: neuroprotective and neurotrophic cells in the central nervous system. Curr. Drug Targets Cardiovasc. and Haematological Disorders 4 (1), 65–84. 10.2174/1568006043481284 15032653

[B44] Nawaz J. Rasul A. Shah M. A. Hussain G. Riaz A. Sarfraz I. (2020). Cardamonin: a new player to fight cancer *via* multiple cancer signaling pathways. Life Sciences 250, 117591. 10.1016/j.lfs.2020.117591 32224026

[B45] Ni H. Li J. Zheng J. Zhou B. (2022). Cardamonin attenuates cerebral ischemia/reperfusion injury by activating the HIF-1α/VEGFA pathway. Phytotherapy Research PTR 36 (4), 1736–1747. 10.1002/ptr.7409 35142404

[B46] Nishal S. Phaugat P. Bazaad J. Dhaka R. Khatkar S. Khatkar A. (2023). A concise review of common plant-derived compounds as a potential therapy for Alzheimer's Disease and Parkinson's Disease: insight into structure-activity-relationship. CNS and Neurological Disorders Drug Targets 22 (7), 1057–1069. 10.2174/1871527321666220614110616 35702799

[B47] Oda K. Matsuoka Y. Funahashi A. Kitano H. (2005). A comprehensive pathway map of epidermal growth factor receptor signaling. Mol. Systems Biology 1, 2005.0010. 10.1038/msb4100014 16729045 PMC1681468

[B48] Peng Y. J. Lu J. W. Lee C. H. Lee H. S. Chu Y. H. Ho Y. J. (2021). Cardamonin attenuates inflammation and oxidative stress in Interleukin-1β-Stimulated osteoarthritis chondrocyte through the Nrf2 pathway. Antioxidants Basel, Switz. 10 (6), 862. 10.3390/antiox10060862 PMC8227809 34072123

[B49] Peng S. Hou Y. Yao J. Fang J. (2017). Activation of Nrf2-driven antioxidant enzymes by cardamonin confers neuroprotection of PC12 cells against oxidative damage. Food and Function 8 (3), 997–1007. 10.1039/c7fo00054e 28271112

[B50] Qin X. Li B. Hu B. Huang J. Tian X. Zhang X. (2024). Rhein inhibits M1 polarization of BV2 microglia through MAPK/IκB signalling pathway and reduces neurotoxicity caused by neuroinflammation. Int. Journal Developmental Neuroscience The Official Journal Int. Soc. Dev. Neurosci. 84 (6), 533–545. 10.1002/jdn.10352 38858813

[B51] Qu W. S. Liu J. L. Li C. Y. Li X. Xie M. J. Wang W. (2015). Rapidly activated epidermal growth factor receptor mediates lipopolysaccharide-triggered migration of microglia. Neurochem. International 90, 85–92. 10.1016/j.neuint.2015.07.007 26209152

[B52] Ru J. Li P. Wang J. Zhou W. Li B. Huang C. Li P. (2014). TCMSP: a database of systems pharmacology for drug discovery from herbal medicines. J. Cheminformatics 13. 10.1186/1758-2946-6-13 24735618 PMC4001360

[B53] Sabbah D. A. Hajjo R. Sweidan K. (2020). Review on epidermal growth factor receptor (EGFR) structure, signaling pathways, interactions, and recent updates of EGFR inhibitors. Curr. Topics Medicinal Chemistry 20 (10), 815–834. 10.2174/1568026620666200303123102 32124699

[B54] Saponaro C. Cianciulli A. Calvello R. Dragone T. Iacobazzi F. Panaro M. A. (2012). The PI3K/Akt pathway is required for LPS activation of microglial cells. Immunopharmacol. Immunotoxicology 34 (5), 858–865. 10.3109/08923973.2012.665461 22397361

[B55] Shie F. S. Chen Y. H. Chen C. H. Ho I. K. (2011). Neuroimmune pharmacology of neurodegenerative and mental diseases. J. Neuroimmune Pharmacology The Official Journal Soc. NeuroImmune Pharmacol. 6 (1), 28–40. 10.1007/s11481-010-9241-8 20820930

[B56] Siddika T. Balasuriya N. Frederick M. I. Rozik P. Heinemann I. U. O'Donoghue P. (2022). Delivery of active AKT1 to human cells. Cells 11 (23), 3834. 10.3390/cells11233834 PMC9738475 36497091

[B57] Smail S. W. (2024). Targeting neuroinflammation and apoptosis: cardamonin's cognitive benefits in alzheimer's 5XFAD mice. Neurochem. Research 50 (1), 57. 10.1007/s11064-024-04308-4 39673650

[B58] Stambolic V. Woodgett J. R. (2006). Functional distinctions of protein kinase B/Akt isoforms defined by their influence on cell migration. Trends Cell Biology 16 (9), 461–466. 10.1016/j.tcb.2006.07.001 16870447

[B59] Stelzer G. Rosen N. Plaschkes I. Zimmerman S. Twik M. Fishilevich S. (2016). The GeneCards suite: from gene data mining to disease genome sequence analyses. Curr. Protocols Bioinformatics 54, 1.30.1–1.3. 10.1002/cpbi.5 27322403

[B60] Stephenson J. Nutma E. van der Valk P. Amor S. (2018). Inflammation in CNS neurodegenerative diseases. Immunology 154 (2), 204–219. 10.1111/imm.12922 29513402 PMC5980185

[B61] Streit W. J. Conde J. R. Fendrick S. E. Flanary B. E. Mariani C. L. (2005). Role of microglia in the central nervous system's immune response. Neurological Research 27 (7), 685–691. 10.1179/016164105X49463 16197805

[B62] Subhramanyam C. S. Wang C. Hu Q. Dheen S. T. (2019). Microglia-mediated neuroinflammation in neurodegenerative diseases. Seminars Cell and Developmental Biology 94, 112–120. 10.1016/j.semcdb.2019.05.004 31077796

[B63] Sun S. C. Ganchi P. A. Ballard D. W. Greene W. C. (1993). NF-kappa B controls expression of inhibitor I kappa B alpha: evidence for an inducible autoregulatory pathway. Science 259 (5103), 1912–1915. 10.1126/science.8096091 8096091

[B64] Temviriyanukul P. Lertmongkolaksorn T. Supasawat P. Pitchakarn P. Thiyajai P. Nusuetrong P. (2022). Phikud navakot extract attenuates lipopolysaccharide-induced inflammatory responses through inhibition of ERK1/2 phosphorylation in a coculture system of microglia and neuronal cells. J. Ethnopharmacology 296, 115440. 10.1016/j.jep.2022.115440 35671865

[B65] Vergadi E. Ieronymaki E. Lyroni K. Vaporidi K. Tsatsanis C. (2017). Akt signaling pathway in macrophage activation and M1/M2 polarization. J. Immunology Baltim. Md 1950 198 (3), 1006–1014. 10.4049/jimmunol.1601515 28115590

[B66] Vogiatzi P. Giordano A. (2007). Following the tracks of AKT1 gene. Cancer Biology and Therapy 6 (10), 1521–1524. 10.4161/cbt.6.10.4834 17921701

[B67] Wang X. McCullough K. D. Franke T. F. Holbrook N. J. (2000). Epidermal growth factor receptor-dependent Akt activation by oxidative stress enhances cell survival. J. Biological Chemistry 275 (19), 14624–14631. 10.1074/jbc.275.19.14624 10799549

[B68] Woodling N. S. Andreasson K. I. (2016). Untangling the web: toxic and protective effects of neuroinflammation and PGE2 signaling in Alzheimer's disease. ACS Chemical Neuroscience 7 (4), 454–463. 10.1021/acschemneuro.6b00016 26979823 PMC5239037

[B69] Wu M. Y. Yang R. S. Lin T. H. Tang C. H. Chiu Y. C. Liou H. C. (2013). Enhancement of PLGF production by 15-(S)-HETE *via* PI3K-Akt, NF-κB and COX-2 pathways in rheumatoid arthritis synovial fibroblast. Eur. Journal Pharmacology 714 (1-3), 388–396. 10.1016/j.ejphar.2013.07.010 23872401

[B70] Wu Y. Wang Y. Liu H. Hu Q. Xie Y. Nan X. (2024). Mechanism of apoptosis in oral squamous cell carcinoma promoted by cardamonin through PI3K/AKT signaling pathway. Sci. Reports 14 (1), 20802. 10.1038/s41598-024-71817-1 PMC11379709 39242879

[B71] Wu M. Chen C. Ren D. Du Z. Liu Z. Liu W. (2025). Cardamonin induces apoptosis of colorectal cancer cells *via* targeted inhibition of the JAK/STAT3/epithelial-mesenchymal transition (EMT) signaling axis. Front. Pharmacology 16, 1739201. 10.3389/fphar.2025.1739201 PMC12823903 41585878

[B72] Yang X. P. Fu J. Y. Yang R. C. Liu W. T. Zhang T. Yang B. (2016). EGFR transactivation contributes to neuroinflammation in Streptococcus suis meningitis. J. Neuroinflammation 13 (1), 274. 10.1186/s12974-016-0734-0 27756321 PMC5070219

[B73] Yang X. Lou Y. Liu G. Wang X. Qian Y. Ding J. (2017). Microglia P2Y6 receptor is related to Parkinson's disease through neuroinflammatory process. J. Neuroinflammation 14 (1), 38. 10.1186/s12974-017-0795-8 28219441 PMC5319038

[B74] Yang T. X. Zhu Y. F. Wang C. C. Yang J. Y. Xue C. H. Huang Q. R. (2022). EPA-enriched plasmalogen attenuates the cytotoxic effects of LPS-stimulated microglia on the SH-SY5Y neuronal cell line. Brain Research Bulletin 186, 143–152. 10.1016/j.brainresbull.2022.06.002 35728742

[B75] Zenke K. Muroi M. Tanamoto K. I. (2018). AKT1 distinctively suppresses MyD88-depenedent and TRIF-dependent toll-like receptor signaling in a kinase activity-independent manner. Cell. Signalling 43, 32–39. 10.1016/j.cellsig.2017.12.002 29242168

[B76] Zhang W. Xiao D. Mao Q. Xia H. (2023). Role of neuroinflammation in neurodegeneration development. Signal Transduction Targeted Therapy 8 (1), 267. 10.1038/s41392-023-01486-5 PMC10336149 37433768

[B77] Zhang W. Guo Y. Wang Q. Wang K. Zhang H. Chang S. (2025). Integrating network pharmacology, machine learning, and experimental validation to elucidate the mechanism of Cardamonin in treating idiopathic pulmonary fibrosis. Int. Journal Molecular Sciences 27 (1), 249. 10.3390/ijms27010249 PMC12786256 41516127

[B78] Zhao M. Wang J. Zhu S. Wang M. Chen C. Wang L. (2024). Mitochondrion-based organellar therapies for central nervous system diseases. Cell Communication Signaling CCS 22 (1), 487. 10.1186/s12964-024-01843-z PMC11468137 39390521

[B79] Zheng L. Wu S. Jin H. Wu J. Wang X. Cao Y. (2023). Molecular mechanisms and therapeutic potential of icariin in the treatment of Alzheimer's disease. Phytomedicine International Journal Phytotherapy Phytopharmacology 116, 154890. 10.1016/j.phymed.2023.154890 37229892

[B80] Zhu Q. Tang T. Liu H. Sun Y. Wang X. Liu Q. (2020). Pterostilbene Attenuates cocultured BV-2 microglial inflammation-mediated SH-SY5Y neuronal oxidative injury *via* SIRT-1 Signalling. Oxidative Medicine Cellular Longevity 2020, 3986348. 10.1155/2020/3986348 32831997 PMC7426790

