# Supplementary material for: Cardamonin attenuates SH-SY5Y neuronal apoptosis induced by LPS-activated microglia via modulation of the EGFR/PI3K/AKT1/ERK/NF-κB pathway
Source: Front Pharmacol. 2026 Oct 19;17:1832453. doi: 10.3389/fphar.2026.1832453 (PMC13724989; doi:10.3389/fphar.2026.1832453)
Supplement: Supplementary file 1 [file Table1.docx]

Supplementary Material

# Methods

# 1 SH-SY5Y Cell viability assay

SH-SY5Y cells were seeded in 96-well plates at a density of 5×10³ cells/well and cultured for 24 h. Subsequently, they were treated with CD (16 μM) alone for 24 h, LPS (1 μg/mL) alone for 24 h, or pretreated with CD (4, 8, or 16 μM) for 2 h followed by LPS (1 μg/mL) stimulation for further 24 h. Control cells were treated with DMSO. Following the treatment, cell viability was evaluated using a cell count kit-8 (CCK-8, Cat: P-CA-001, Pricella, Wuhan, China). The absorbance at 450 nm was measured using a spectrophotometer (Thermo Scientific Multiskan Skylitch, Type 1550, USA).

# 2 Assessment of morphological changes

SH-SY5Y cells (3.5×10⁵ cells/well) were incubated overnight in 6-well plates at 37℃.The cells were then treated with CD (16 μM) alone for 24 h, LPS (1 μg/mL) alone for 24 h, or pretreated with CD (4, 8, or 16 μM) for 2 h followed by LPS (1 μg/mL) stimulation for further 24 h. Control cells were treated with DMSO. The morphological changes were observed and photographed using EOS Utility Software (Canon USA, Inc).

**Figure captions**

Supplementary Figure 1 Direct effects of LPS and CD on SH-SY5Y cell viability and morphology. (A) Cell viability measured by CCK-8 assay. (B) Representative light microscopy images showing morphological changes (10X). Data were presented as the mean ± SD (n=6). ^####^p<0.0001, ^###^p < 0.001 versus the control group.
